# Direct impact of commonly used dietary emulsifiers on human gut microbiota

**DOI:** 10.1186/s40168-020-00996-6

**Published:** 2021-03-22

**Authors:** Sabrine Naimi, Emilie Viennois, Andrew T. Gewirtz, Benoit Chassaing

**Affiliations:** 1 INSERM U1016, team “Mucosal microbiota in chronic inflammatory diseases”, CNRS UMR 8104, Université de Paris, Paris, France; 2 INSERM, U1149, Center of Research on Inflammation, Université de Paris, Paris, France; 3 Institute for Biomedical Sciences, Center for Inflammation, Immunity and Infection, Digestive Disease Research Group, Georgia State University, Atlanta, GA USA

**Keywords:** Gut microbiota, Intestinal inflammation, IBD, Dietary emulsifier

## Abstract

**Background:**

Epidemiologic evidence and animal studies implicate dietary emulsifiers in contributing to the increased prevalence of diseases associated with intestinal inflammation, including inflammatory bowel diseases and metabolic syndrome. Two synthetic emulsifiers in particular, carboxymethylcellulose and polysorbate 80, profoundly impact intestinal microbiota in a manner that promotes gut inflammation and associated disease states. In contrast, the extent to which other food additives with emulsifying properties might impact intestinal microbiota composition and function is not yet known.

**Methods:**

To help fill this knowledge gap, we examined here the extent to which a human microbiota, maintained ex vivo in the MiniBioReactor Array model, was impacted by 20 different commonly used dietary emulsifiers. Microbiota density, composition, gene expression, and pro-inflammatory potential (bioactive lipopolysaccharide and flagellin) were measured daily.

**Results:**

In accordance with previous studies, both carboxymethylcellulose and polysorbate 80 induced a lasting seemingly detrimental impact on microbiota composition and function. While many of the other 18 additives tested had impacts of similar extent, some, such as lecithin, did not significantly impact microbiota in this model. Particularly stark detrimental impacts were observed in response to various carrageenans and gums, which altered microbiota density, composition, and expression of pro-inflammatory molecules.

**Conclusions:**

These results indicate that numerous, but not all, commonly used emulsifiers can directly alter gut microbiota in a manner expected to promote intestinal inflammation. Moreover, these data suggest that clinical trials are needed to reduce the usage of the most detrimental compounds in favor of the use of emulsifying agents with no or low impact on the microbiota.

**Video abstract**

**Supplementary Information:**

The online version contains supplementary material available at 10.1186/s40168-020-00996-6.

## Background

The gastro-intestinal tract is colonized by a vast complex community of microorganisms including bacteria, viruses, protozoa, and fungi, collectively referred to as the gut microbiota. The gut microbiota plays important physiologic roles, especially in terms of mediating metabolism, driving host immune system development, and impeding infection by pathogens. However, accumulating evidence demonstrate that detrimental alterations in microbiota, loosely referred to as dysbiosis, can promote chronic inflammatory diseases, such as metabolic syndrome and inflammatory bowel disease (IBD), whose main forms include Crohn’s disease and ulcerative colitis [1, 2]. More specifically, lasting disturbance of the microbiota can result in chronic intestinal inflammation that promotes the development of these disorders [3, 4]. While various factors have potential to alter intestinal microbiota, dietary components, especially food additives whose advent associates with the post-mid twentieth century increase non-infectious inflammatory diseases, are particularly suspicious [5, 6]. Such food additives are frequently non-absorbed and thus will likely directly interact with the microbiota. Several studies have reported that artificial sweeteners and polysaccharides can promote intestinal inflammation and metabolic dysregulation [7–10]. Another example of this concept is our observation that dietary emulsifiers can promote chronic intestinal inflammation in mice [5, 11]. Emulsifiers are chemicals that enable homogenization of immiscible liquids and are incorporated into many processed foods in order to improve texture and extend shelf life [12]. While the limited testing of food additives indicates that these compounds generally lack over toxicity and are not mutagenic, there is, nonetheless, considerable basis to question their safety, particularly in the context of chronic inflammatory diseases. For example, carrageenan induces chronic intestinal inflammation in rodents [13], and our studies of carboxymethylcellulose (CMC, E466) and polysorbate 80 (P80, E433) revealed that these compounds detrimentally alter intestinal microbiota composition and function, in ways that promoted chronic intestinal inflammation [5]. Mechanistically, we found, using mice and in vitro models, that intestinal microbiota is a direct target of CMC and P80. Moreover, germfree animals are completely protected against CMC- and P80-induced inflammation, while in vitro microbiota treated with CMC or P80 are detrimentally impacted in ways that can lead to chronic intestinal inflammation when transferred to germfree recipient animals [5, 11, 14]. Such studies indicate the central role of direct microbiota disturbances in mediating the detrimental impacts of CMC and P80.

Recent studies questioning food additive safety has prompted the launch of multiple clinical trials aiming to investigate the impact of these compounds on human health [15–17]. Furthermore, these studies highlight that safety evaluation of a food additive, especially non-absorbed compounds, should consider impacts on gut microbiota. Hence, as practical means of testing such food additives, we used an in vitro model, namely the MiniBioReactor Arrays (MBRA) [18, 19] which allow dynamic stable culture of human-derived microbiota under anaerobic conditions, to evaluate the impact of 20 widely used dietary emulsifiers on human microbiota. In accord with previous studies, both CMC and P80 significantly impact microbiota composition and function during the treatment as well as in the post-treatment phase, suggesting that the detrimental effects of these compounds are long-lasting. While similar results were obtained with most of the other 18 compounds tested, some had minimal impacts on the microbiota in this system, suggesting that these particular emulsifiers may lack the potential to promote chronic inflammation.

## Methods

### MiniBioReactor Arrays (MBRAs) operation

#### Fecal sample collection

Fecal samples from a healthy individual were collected into sterile containers, sealed, and transferred to an anaerobic chamber within 10 min of defecation. The fecal sample was manually homogenized and subdivided into sterile 50-mL tubes and stored at – 80 °C until use. The research protocol was approved by the GSU IRB committee under approval number H19174. Individual donating samples provided informed consent prior to donation.

#### MBRA set-up, inoculation, and sample collection

MBRAs were prepared as previously described [18] and as presented in Figure S1. Briefly, this system, housed in an anaerobic chamber, consisted of 24 individual chambers containing 15 mL of Bioreactor Medium (BRM), as described in [18], except that polysorbate 80 (P80) was removed in order to have an emulsifier-free medium, and the 1 g/L of taurocholic acid was replaced with 0.5 g/L of bovine bile added prior to autoclaving. MBRA chambers were held on a magnetic stand for continual homogenization and were connected to two 24-channel peristaltic pumps with low flow-rate capabilities (205S peristaltic pump with 24-channel drive, Watson-Marlow). After autoclaving of the MBRA chambers and tubing, the system was put in place and left in the anaerobic chamber for at least 48 h. Chambers were filled with BRM and subsequently inoculated. For MBRA inoculation, fecal samples were resuspended at 25% w/v in anaerobic phosphate-buffered saline in the anaerobic chamber, vortexed for 5 min, and centrifuged at 200*g* for 5 min. The supernatant was subsequently collected in the anaerobic chamber and filtered through a 100-μm filter to remove any particles. 3.8 mL of this fecal slurry was used to inoculate each MBRA chamber. After inoculation, fecal bacteria were allowed to equilibrate for 16 h prior to initial sample collection (time 0 sample, Figure S1C) and flow initiation at 1.875 mL/h (8-h retention time). Four hundred microliters of samples was then collected as presented in Figure S1C (0 h, 24 h, 48 h, 72 h, 74 h, 77 h, 80 h, 96 h, 108 h, 120 h, 144 h, 168 h, 192 h, 216 h, 240 h, 264 h, and 274 h), with emulsifier treatment occurring between 72 and 216 h post-inoculation, as described below. Samples were stored at − 80 °C until further processed.

#### Treatment with emulsifying agents

As presented in Table 1 and Figure S1, 20 food additives with emulsifying properties were used in this study. Three independent experiments were performed, as presented in Figure S1C, with each experiment containing control (untreated) chambers and using the same fecal samples from the same donor. Emulsifying agents were added to the BRM medium prior to autoclaving at a concentration of 0.1%. Seventy-two hours after inoculation and use of BRM medium, bottles containing emulsifying agents were connected to the system and use to feed the chambers from 72- to 216-h time points, at which time emulsifier-free BRM medium was connected back to the system. Each condition was performed in triplicate.

**Table 1:** List of the twenty emulsifiers used

Emulsifier agent	E number	Manufacturer
Sodium carboxymethylcellulose (CMC, average MW~250,000)	466	Sigma (Sigma, St. Louis, MO, USA)
Polysorbate 80 (P80)	433	Sigma (Sigma, St. Louis, MO, USA)
Soy lecithin	322	Modernist Pantry LLC (Eliot, ME, USA)
Sunflower lecithin	322	Swanson Health Products (Fargo, ND, USA)
Maltodextrin	1400	Bulk Supplements (Henderson, NV, USA)
Propylene glycol alginate	405	Modernist Pantry LLC (Eliot, ME, USA)
Iota carrageenan	407	Modernist Pantry (York, ME, USA)
Kappa carrageenan	407	Modernist Pantry (York, ME, USA)
Lambda carrageenan	407	Modernist Pantry LLC (Eliot, ME, USA)
Xantham gum	415	Judee's Gluten Free (OH, USA)
Gum arabic	414	Frontier Co-op (Norway, IA, USA)
Guar gum	412	Now Foods (Bloomingdale, IL, USA)
Locust bean gum	410	Modernist Pantry LLC (Eliot, ME, USA)
Agar agar	406	ScrapCooking (France)
Diacetyl tartaric acid ester of mono- and diglycerides (DATEM)	472e	Modernist Pantry LLC (Eliot, ME, USA)
Hydroxypropyl methyl cellulose (HPMC, average MW~90,000)	464	Sigma (Sigma, St. Louis, MO, USA)
Sorbitan monostearate	491	Sigma (Sigma, St. Louis, MO, USA)
Mono- and diglycerides	471	Modernist Pantry LLC (Eliot, ME, USA)
Glyceryl Stearate	471	Lonza Inc (Allendale, NJ, USA)
Glyceryl Oleate	471	Lonza Inc (Allendale, NJ, USA)

### Materials

Food additives used in this study are listed in Table 1.

### Bacterial DNA extraction

DNA was extracted from 200-μL frozen MBRA suspension using a QIAamp 96 PowerFecal QIAcube HT kit from Qiagen Laboratories (Venlo, Netherlands) with mechanical disruption (bead-beating). Briefly, 650 μL of prewarmed buffer PW1 was added to 50 μL of each sample. Samples were thoroughly homogenized using bead-beating with a TissueLyser before centrifuging the plate at 4000 rpm for 5 min at 20 °C in order to pellet beads and particles. Four hundred microliters of supernatant was added into a new 96-well plate containing 150 μL of Buffer C3. After mixing and incubation on ice for 5 min, centrifugation was performed at 4000 rpm for 5 min at 20 °C. Three hundred microliters of each supernatant was added to a new 96-well plate, and 20 μL of Proteinase K was added and incubated for 10 min at room temperature. The following steps were next performed on a QIAcube high-throughput robot: addition of 500 μL of Buffer C4, DNA binding to a QIAamp 96 plate, column wash using AW1 (800 μL), AW2 (600 μL), and ethanol (400 μL), and DNA elution using ATE buffer (100 μL).

### Bacterial density quantification by 16S rRNA qPCR

Extracted DNAs were diluted 1/10 with sterile DNA-free water and amplified by quantitative PCR using the 16S V4 specific primers 515F 5′-GTGYCAGCMGCCGCGGTAA-3′ and 806R 5′-GGACTACNVGGGTWTCTAAT-3′ on a Biorad CFX96 apparatus (BioRad) using QuantiFast SYBR® Green PCR Kit (Qiagen). Amplification of a single expected PCR product was confirmed by electrophoresis on a 2% agarose gel.

### Microbiota analysis by 16S rRNA gene sequencing using Illumina technology

16S rRNA gene amplification and sequencing were done using the Illumina MiSeq technology following the protocol of the Earth Microbiome Project with some slight modifications (www.earthmicrobiome.org/emp-standard-protocols) [20, 21]. Briefly, the 16S rRNA genes, region V4, were PCR amplified from each sample using a composite forward primer and a reverse primer containing a unique 12-base barcode, designed using the Golay error-correcting scheme, which was used to tag PCR products from respective samples [21]. We used the forward primer 515F 5′-*AATGATACGGCGACCACCGAGATCTACACGCT*XXXXXXXXXXXX**TATGGTAA TT*GT***GTGYCAGCMGCCGCGGTAA-3′: the italicized sequence is the 5′ Illumina adapter, the 12 X sequence is the Golay barcode, the bold sequence is the primer pad, the italicized and bold sequence is the primer linker, and the underlined sequence is the conserved bacterial primer 515F. The reverse primer 806R used was 5′-*CAAGCAGAAGACGGCATACGAGAT***AGTCAGCCAG** ***CC***GGACTACNVGGGTWTCTAAT-3′: the italicized sequence is the 3′ reverse complement sequence of Illumina adapter, the bold sequence is the primer pad, the italicized and bold sequence is the primer linker, and the underlined sequence is the conserved bacterial primer 806R. PCR reactions consisted of 5PRIME HotMasterMix (Quantabio, Beverly, MA, USA), 0.2 μM of each primer, 10–100 ng template, and reaction conditions were 3 min at 95 °C, followed by 30 cycles of 45 s at 95 °C, 60 s at 50 °C, and 90 s at 72 °C on a Biorad thermocycler. PCR products were visualized by gel electrophoresis and purified with Ampure magnetic purification beads (Agencourt, Brea, CA, USA). Products were then quantified (BIOTEK Fluorescence Spectrophotometer), and a master DNA pool was generated from the purified products in equimolar ratios. The pooled products were quantified using Quant-iT PicoGreen dsDNA assay and sequenced using an Illumina MiSeq sequencer (paired-end reads, 2 × 250 bp) at Cornell University, Ithaca.

### 16S rRNA gene sequence analysis

16S rRNA sequences were analyzed using QIIME2—version 2019 [22]. Sequences were demultiplexed and quality filtered using the Dada2 method [23] with QIIME2 default parameters in order to detect and correct Illumina amplicon sequence data, and a table of Qiime 2 artifact was generated. A tree was next generated, using the align-to-tree-mafft-fasttree command, for phylogenetic diversity analyses, and alpha and beta diversity analyses were computed using the core-metrics-phylogenetic command. Principal coordinate analysis (PCoA) plots were used to assess the variation between the experimental group (beta diversity). For taxonomy analysis, features were assigned to operational taxonomic units (OTUs) with a 99% threshold of pairwise identity to the Greengenes reference database 13_8 [24]. Unprocessed sequencing data are deposited in the Genome Sequence Archive (GSA) in BIG Data Center, Beijing Institute of Genomics, Chinese Academy of Sciences, under accession number CRA005149, publicly accessible at http://bigd.big.ac.cn/gsa.

### Fecal flagellin and lipopolysaccharide load quantification

Levels of fecal bioactive flagellin and lipopolysaccharide (LPS) were quantified as previously described [25] using human embryonic kidney (HEK)-Blue-mTLR5 and HEK-BluemTLR4 cells, respectively (Invivogen, San Diego, CA, USA) [25]. MBRA samples (whole suspension, without centrifugation) were serially diluted and applied on mammalian cells. Purified *E. coli* flagellin and LPS (Sigma-Aldrich) were used for standard curve determination using HEK-Blue-mTLR5 and HEK-Blue-mTLR4 cells, respectively. After 24 h of stimulation, the cell culture supernatant was applied to QUANTI-Blue medium (Invivogen) and the alkaline phosphatase activity was measured at 620 nm after 30 min.

### Metatranscriptomic analysis

Total RNAs were extracted from MBRA suspension collected in the middle of the treatment phase (120-h time point, selected to not miss any transient impact of select emulsifiers on the microbiota) selected in using RNeasy PowerMicrobiome Kit (Qiagen), according to the manufacturer’s protocol. After purification, RNA concentration and integrity were determined using Epoch Microplate Spectrophotometer (Bio-Tek, Winooski, Vermont, USA) and agarose gel electrophoresis, respectively. Total RNA was then prepared for sequencing using KAPA Stranded mRNA-seq kit and according to the manufacturer’s protocol. Briefly, rRNA were depleted using QiaSeq FastSelect rRNA Removal kit (Qiagen) according to the manufacturer’s protocol for ribodepletion. rRNA-depleted RNAs were fragmented and converted to cDNA. After end repair and ligation of adapters, mRNA libraries were amplified by PCR and validated using BioAnalyser, according to the manufacturer’s recommendations. The purified library was then subjected to sequencing using an Illumina NextSeq500 at the Georgia Genomics and Bioinformatics Core (https://dna.uga.edu/, Athens, GA, USA). Sequencing data obtained were demultiplexed and quality filtered using fastq_quality_filter command (http://hannonlab.cshl.edu/fastx_toolkit/) with -q28 -p 80 in order to only keep sequences with at least 80% of bases having a minimum quality score of 28. Nest, SortMeRNA version 2.1 (https://bioinfo.lifl.fr/RNA/sortmerna/) was used in order to remove rRNA sequences using the following reference databases: silva-arc-16 s-id95.fasta, silva-arc-23 s-id98.fasta, silva-bac-16 s-id90.fasta, silva-bac-23 s-id98.fasta, silva-euk-18 s-id95.fasta, silva-euk-28 s-id98.fasta, rfam-5 s-database-id98.fasta, and rfam-5.8 s-database-id98.fasta. The obtained quality-filtered and rRNA-filtered sequence file was uploaded to Galaxy Europe https://usegalaxy.eu/ and used to run HUMAnN2 program in order to profile for the abundance of microbial pathways and gene families [26]. A transcript table with relative abundance was generated for each sample and compared using either principal coordinate analysis of the Bray-Curtis distance or volcano plot created using R software. Unprocessed sequencing data are deposited in the Genome Sequence Archive (GSA) in BIG Data Center, Beijing Institute of Genomics, Chinese Academy of Sciences, under accession number CRA005148, publicly accessible at http://bigd.big. ac.cn/gsa.

### Data presentation and statistical analysis

Data are presented as the means ± S.E.M. For bacterial density, alpha and beta diversity analysis of microbiota composition and expression of pro-inflammatory molecules, in order to account for the high number of time points analyzed (17) during the three phases of the MBRA experiments (pre-treatment, treatment and post-treatment), data presented as fully detailed in Figure S2. As exemplified for the Jaccard measurement of beta diversity of microbiota composition, we first performed principal coordinate analysis as each individual time point (Figure S2A). We next used these analyses to plot histograms of Jaccard distance separating control samples from every other condition—including control themselves (Figure S2B). Such presentation allows, for each time point, to see the alterations in microbiota composition induced by emulsifier exposure. These various time points were subsequently combined in a XY representation with two normalizations being applied:

- In the left graph panel S2C, the distance separating control samples from themselves was determined as 1, for each specific time point, in order to account for time point to time point variations (Figure S2C). Hence, such representation results in a flat control curve with an average of 1 at each time point.

- In the right graph panel S2C, the obtained relative values from the left graph were normalized to 1 at the 24-h time point, for each specific treatment, in order to account for pre-treatment/basal variations in microbiota composition and function, inherent to the MBRA system used (Figure S2C). Hence, such representation results in all curves having an average of 1 at the 24-h time point.

As presented in Figure S2D, such data presentation allows to normalize based on control chambers as well as based on a pre-treatment time point. Finally, the area under the curve was determined for the treatment phase (72 h > 216 h) and the post-treatment phase (216 h > 274 h) in order to present, for both phases, the global impact of emulsifier exposure on microbiota composition and function. For bacterial density and alpha diversity analysis, since emulsifier exposure can lead to a significant reduction in the indexes used, the same approach was used to calculate both the area under the curve above and below the baseline (1, represented by control samples). Such presentation importantly allows to not rely on one single time point for microbiota composition and function assessment, but instead, to account for all the time points collected during a specific phase (treatment and post-treatment). Moreover, values obtained at the 96-h, 216-h, and 274-h time points are presented Figure S3 (bacterial density), Figure S4 (beta diversity analysis of microbiota composition), Figure S5 (alpha diversity analysis of microbiota composition), and Figure S6 (expression of pro-inflammatory molecules).

Significance was determined using one-way group ANOVA with Bonferroni’s multiple comparisons test (GraphPad Prism software, version 6.01). Differences were noted as significant *p ≤ 0.05.

## Results

### Several dietary emulsifiers impact microbiota density in the MBRA model

**Fig. 1:**
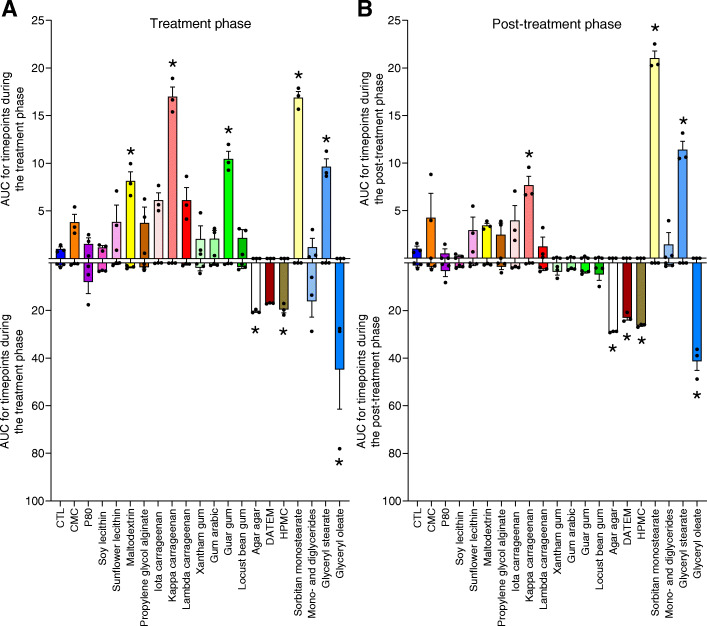
Impact of dietary emulsifiers on bacterial density. Bacterial density was assessed by qPCR during the treatment (**a**) and the post-treatment phases (**b**). Area under curve (AUC) was calculated for both increase and decrease in bacterial density, as detailed in Figure S2 and in the “Methods” section. Data are the means ± S.E.M, with individual data points being represented (*N =* 3). *P < 0.05 compared to the untreated group, determined by a one-way analysis of variance corrected for multiple comparisons with a Bonferroni post-test

Two widely used synthetic dietary emulsifiers, CMC and P80, directly alter the composition and gene expression of human microbiota in vitro, in doses ranging from 0.10 to 1.00%, recapitulating their actions in vivo in animal models [5, 11]. We sought to investigate if such impacts on microbiota were unique to these emulsifiers or reflected a general characteristic of this class of food additive. Hence, we examined the impact of 17 other commonly used dietary emulsifiers (Table 1) on parallel anaerobic human microbiota cultures using the MBRA system, as outlined in Figure S1. We also tested the impact of a broadly used food additive with emulsifier properties, namely the texture-enhancer maltodextrin [12]. Three replicate experiments were performed, with each one testing 6–7 emulsifiers in triplicate, together with 3 control (i.e., untreated) microbiota cultures, all of which were generated from a single healthy subject. Samples were collected at 17 different time points (4 pre-treatments, 10 during treatment, 3 post-treatment), as outlined in Figure S1. Emulsifying agents were added to the BRM medium prior to autoclaving at a concentration of 0.1%, based on our previous studies in mice investigating CMC and P80 [5, 11, 27], as well as estimated consumption in human [12]. We first measured the impact of these compounds on microbiota density, as measured by qPCR using universal 16S primers. As detailed in the “Methods” section and presented in Fig. 1, results are displayed as the impact of each compound relative to untreated microbiotas during the treatment and post-treatment period. This approach avoided reliance on one single time point and, rather, accounted for data from all the time points collected during a specific phase (treatment and post-treatment). Such approach identified that 2 emulsifiers, namely glycerol stearate and sorbitan monostearate, as well as carrageenans, increased bacterial density during the treatment and post-treatment phases while guar gum and maltodextrin significantly increased this parameter only during the treatment phase. In contrast, agar, DATEM, HPMC, and glyceryl oleate led to an irreversible decrease in microbial bacterial density. Thus, some, but not all, commonly used dietary emulsifiers can alter the overall levels of bacteria in the gut microbiota.

### Most, but not all, emulsifiers impact microbiota composition

**Fig. 2:**
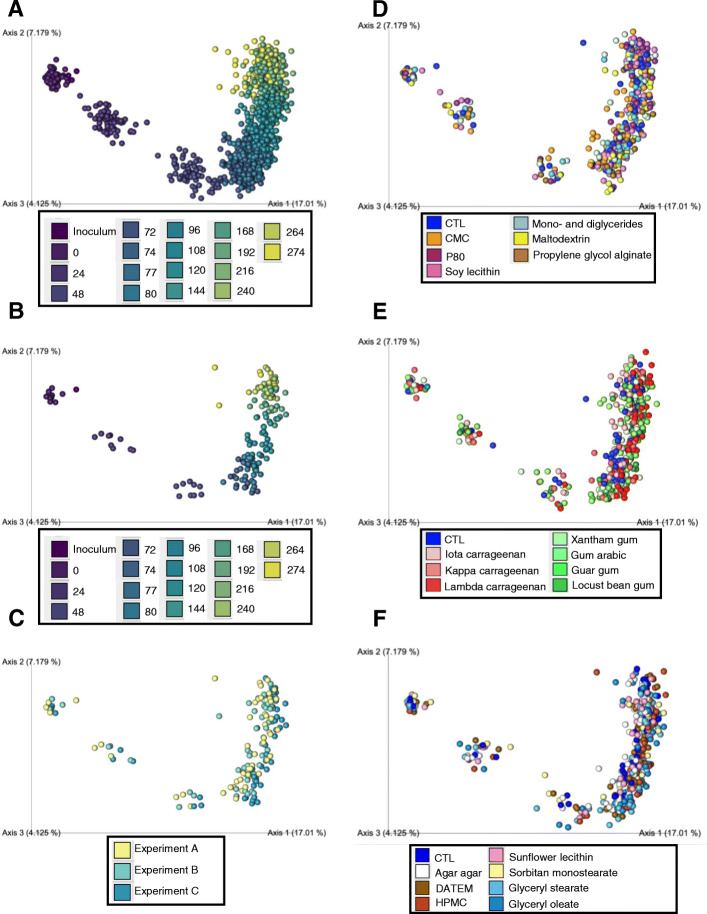
Impact of dietary emulsifiers on microbiota composition. Microbiota composition was analyzed by 16S rRNA gene sequencing and beta diversity was computed through QIIME2 pipeline using the Jaccard matrix. **a** Principal coordinate analysis (PCoA) of the Jaccard matrix from all the time points and treatments analyzed from the three independent experiments. Dots are colored by time point. **b**, **c** PCoA of the Jaccard matrix from all the time points of the untreated MBRA chambers from the three independent experiments and colored by time point (**b**) or experiment (**c**). **d**–**f** Principal coordinate analysis (PCoA) of the Jaccard matrix from all the time points and treatments analyzed from each independent experiment. Dots are colored by treatment. *N =* 3

We next investigated the impact of these food additives on microbiota composition via 16S rRNA gene sequencing. Data were first assessed via the use of principal coordinate analysis (PCoA) of the Jaccard matrix from all of the samples generated in the three independent experiments. Color coding based on time point (i.e., irrespective of treatment) revealed a marked shift during the first 48 h, particularly along PC1, which accounted for 17% of the overall differences in the samples, after which time changes were relatively modest and primarily observed along PC2, which accounted for 7.2% of the overall differences between the samples (Fig. 2a). That similar temporal changes were seen when looking at untreated samples indicated that this pattern reflected stabilization of the fecal microbiota in the MBRA system, as previously reported [19] (Fig. 2b). Importantly, color coding of the untreated samples across the 3 replicate experiments demonstrated that the pattern of stabilization was highly reproducible between independent experiments (Fig. 2c). As presented in Figure S1D, stabilization of the MBRA involved an initial drop in alpha diversity within 48 h which remained stable thereafter similarly to previous studies using this or other microbiota models [11, 19]. Taxonomically, microbiota composition was found stable over time after an initial shift during the first 24 h of culture (Figure S7). Importantly, the relative abundance of the orders belonging to the proteobacteria phylum is remaining at a low level during the entire experiment, while other studies have observed a bloom of this phylum when using other in vitro gut system [28], further highlighting the benefit of using the MBRA system as an in vitro microbiota system. Overall, these results demonstrate that MBRA microbiota stability is reached at the 72-h time point at which time we decided to initiate treatment. Hence, this supports our approach of comparing distinct treatments used in 3 non-parallel replicate experiments that comprised this study.

**Fig. 3:**
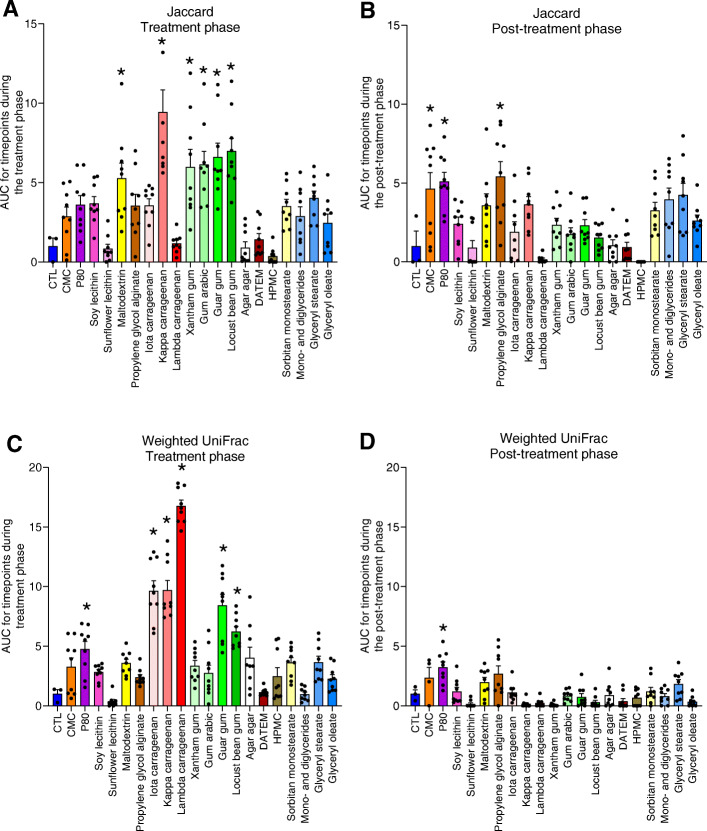
Impact of dietary emulsifiers on microbiota composition. Microbiota composition was analyzed by 16S rRNA gene sequencing and beta diversity was computed through QIIME2 pipeline using the Jaccard matrix (**a**, **b**) and the unweighted UniFrac distance (**c**, **d**) during the treatment (**a**, **c**) and the post-treatment phases (**b**, **d**). Area under curve (AUC) was calculated, as detailed in Figure S2 and in the “Methods” section. Data are the means ± S.E.M, with individual data points being represented (*N =* 3). *P < 0.05 compared to the untreated group, determined by a one-way analysis of variance corrected for multiple comparisons with a Bonferroni post-test

**Fig. 4:**
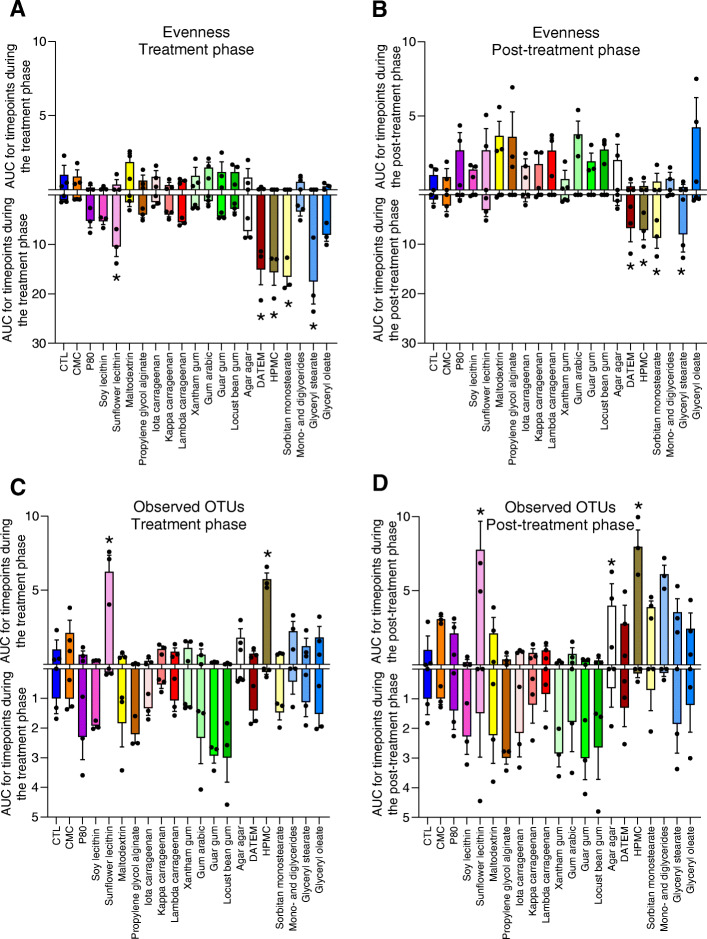
Impact of dietary emulsifiers on microbiota diversity. Microbiota composition was analyzed by 16S rRNA gene sequencing and alpha diversity was computed through QIIME2 pipeline using the evenness index (**a**, **b**) and the number of observed OTUs (**c**, **d**) during the treatment (**a**, **c**) and the post-treatment phases (**b**, **d**). Area under curve (AUC) was calculated for both increase and decrease in alpha diversity, as detailed in Figure S2 and in the “Methods” section. Data are the means ± S.E.M, with individual data points being represented (*N =* 3). *P < 0.05 compared to the untreated group, determined by a one-way analysis of variance corrected for multiple comparisons with a Bonferroni post-test

Color coding of PCoA plots based on the dietary emulsifier were exposed to showed some level of clustering during the treatment phase (Fig. 2d–f) indicating that, indeed, many of these compounds were impacting microbiota composition, although the continuing, albeit diminished, changes in untreated samples during the treatment phase makes such clustering easier to appreciate when looking at individual time points (Figure S2). As a means to quantitate the overall extent to which tested compounds impacted microbiota composition, amidst the continuing changes irrespective of treatment, we quantified the distance separating treated samples from untreated samples, at each time point. Specifically, we calculated both weighted UniFrac and Jaccard matrix distances, which, respectively, consider, or disregard, phylogenetic distance and relative abundance of microbiota members. These analyses, presented in Fig. 3, revealed a significant impact of CMC, P80, maltodextrin, propylene glycol alginate, kappa carrageenan, gum arabic and xantham, guar, and locust bean gums on microbiota composition relative to control samples, as assessed by Jaccard distances (Fig. 3a, b). A generally similar pattern was observed when analyzing this data with the weighted UniFrac metric, which yielded the previously observed impact of P80 [11], but also found that all tested forms of carrageenan, guar gum, and locust bean gum significantly altered microbiota composition (Fig. 3c, d). Interestingly, the limited impact of emulsifiers on the weighted UniFrac distance during the post-treatment phase (Fig. 3d) suggests that the more abundant microbial members are more resilient than the less abundant microbial members, as observed using the unweighted metric (Fig. 3b). All together, these data indicate the broad and heterogeneous ability of specific emulsifiers to affect microbiota composition. Investigation of alpha diversity, using evenness index and the number of observed OTUs, confirmed the heterogenous response (Fig. 4). For example, a significant and non-reversible decrease in evenness was observed in microbiotas exposed to DATEM, HPMC, sorbitan monostearate, and glyceryl stearate (Fig. 4a, b).

**Fig. 5:**
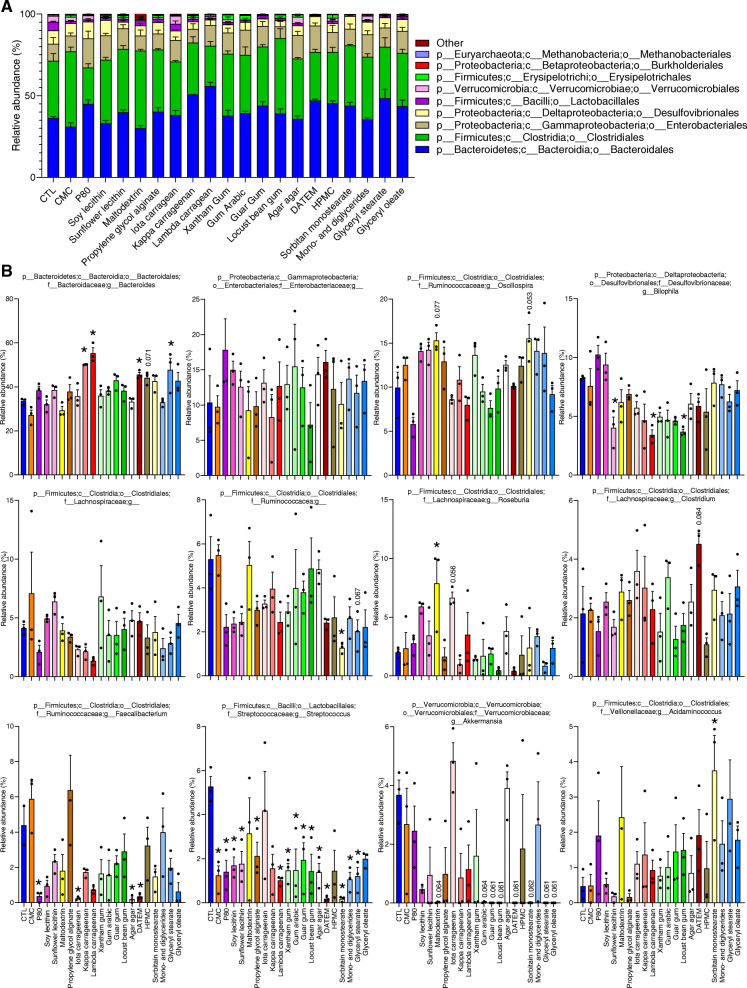
Impact of dietary emulsifiers on microbiota composition at various taxonomic levels. **a** Microbiota composition was analyzed by 16S rRNA gene sequencing and taxonomic analysis were computed through QIIME2 pipeline at the order level (144-h time point). **b** Microbiota composition was analyzed by 16S rRNA gene sequencing and taxonomic analysis were computed through QIIME2 pipeline at the genus level (144-h time point). Only the 12 more abundant genera are represented, from the more abundant (top left) to the least abundant (bottom right). Data are the means ± S.E.M, with individual data points being represented (*N =* 3). *P < 0.05 compared to the untreated group, determined by a one-way analysis of variance corrected for multiple comparisons with a Bonferroni post-test

Taxonomic analysis at the order level, performed on samples collected in the middle of the treatment phase (144 h post-inoculation, 72 h after the beginning of emulsifier exposure), revealed that numerous dietary emulsifiers induced a significant reduction in Lactobacillales, which was mostly driven by a significant reduction in the *Streptococcus* genus (Figs. 5 and S8). Amongst the 12 most abundant genus, *Bacteroides* was significantly enriched by kappa and lambda carrageenans as well as by DATEM and glyceryl stearate, while P80, iota carrageenan, agar agar, and DATEM significantly decreased the relative abundance of *Faecalibacterium*, also known for its anti-inflammatory properties [29] (Fig. 5). Overall, these data indicate that some broadly used food additives significantly impacted microbiota composition in the MBRA model in a manner that can be envisioned to impact function, particularly as relates to ability to dampen/promote inflammation.

### Select emulsifiers increase the expression of microbiota-derived pro-inflammatory molecules

**Fig. 6:**
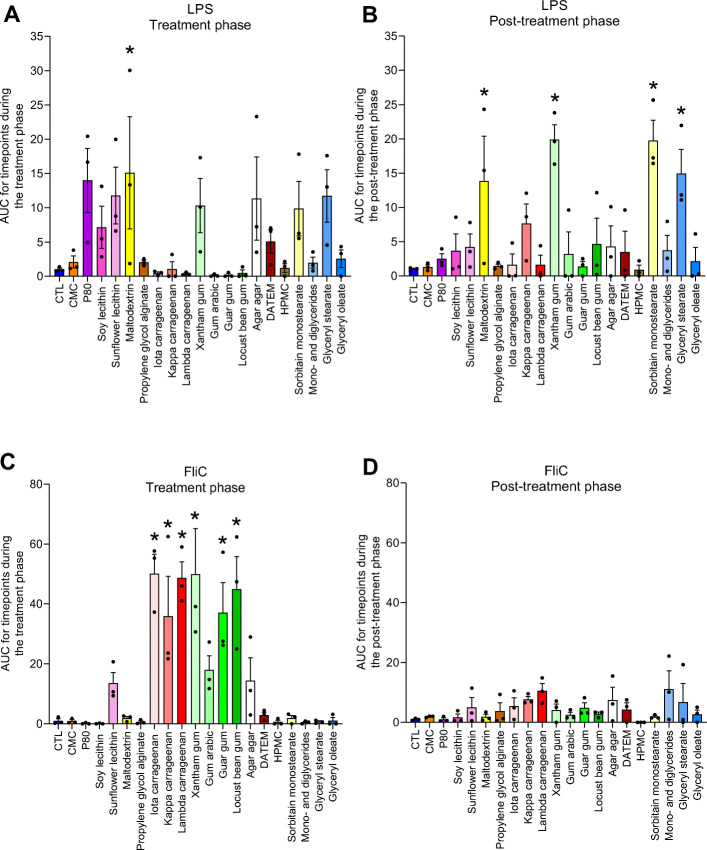
Impact of dietary emulsifiers on the expression of microbiota-derived pro-inflammatory molecules. Microbiota-derived expression of pro-inflammatory molecules was analyzed using HEK cells expressing TLR4 or TLR5 in order to quantify bioactive levels of lipopolysaccharide (LPS) (**a**, **b**) and flagellin (FliC) (**c**, **d**), respectively, during the treatment (**a**, **c**) and the post-treatment phases (**b**, **d**). Area under curve (AUC) was calculated, as detailed in Figure S2 and in the “Methods” section. Data are the means ± S.E.M, with individual data points being represented (*N =* 3). *P < 0.05 compared to the untreated group, determined by a one-way analysis of variance corrected for multiple comparisons with a Bonferroni post-test

Detrimental impacts of CMC and P80 in vivo associate with these compound’s increasing levels of bioactive LPS and flagellin, in vivo and in vitro, thus suggesting a potential means by which impacting microbiota might promote inflammation [5, 14]. Hence, we next examined if levels of these microbial pro-inflammatory agonists were impacted in the MBRA model by the compounds tested here. Levels of bioactive LPS and flagellin were measured via HEK cells engineered to express TLR4 or TLR5, respectively, and specific peroxidase under control of an NF-κB-responsive promoter. These data, presented in Fig. 6, revealed significantly increased LPS levels in microbiota exposed to maltodextrin, which persisted during the post-treatment phase. Microbiotas exposed to xantham gum, sorbitain monostearate, and glyceryl stearate displayed a tendency of increased LPS levels during the treatment phase, which became significant in the post-treatment phase, suggesting that these dietary emulsifiers induce slow but persistent increase in the microbiota’s expression of these pro-inflammatory molecules. Moreover, all carrageenans (iota, kappa, and lambda), as well as xantham gum, guar gum, and locust bean gum, significantly induced bioactive levels of flagellin in a reversible manner (Fig. 6c, d). Interestingly, CMC and P80 had only modest impacts on levels of flagellin and LPS, possibly reflecting a degree of donor-specific resistance to these specific additives. Moreover, as samples were not normalized based on their bacterial density, some of these increases in the expression of pro-inflammatory molecules could be linked to an increase in bacterial load, as observed for maltodextrin, lambda carrageenan, guar gum, sorbitain monostearate, and glyceryl stearate. Nonetheless, overall, these functional microbiota readouts suggest that numerous emulsifiers significantly enhance the ability of the microbiota to activate innate immune signaling pathways thought to contribute to inflammation in the intestinal tract [25].

### Impact of commonly used emulsifiers on human microbiota metatranscriptome

**Fig. 7:**
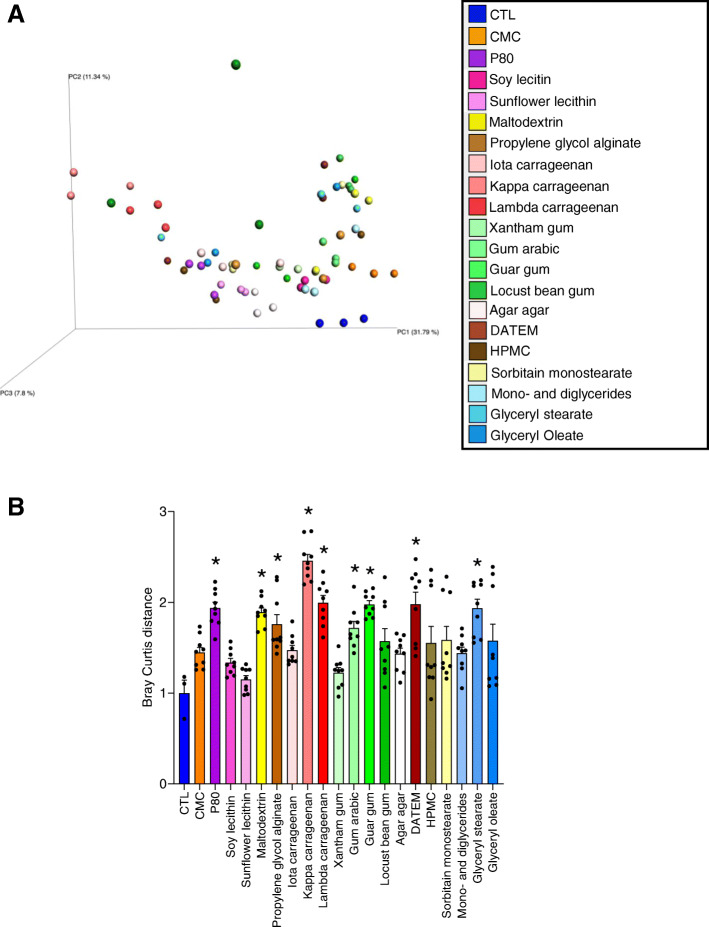
Impact of dietary emulsifiers on in vitro microbiota metatranscriptomes. Total RNAs were extracted from MBRA suspension collected at the 120-h time point and subjected to RNA sequencing. **a** HUMAnN2 program was used to profile for the abundance of microbial pathways and gene families, and a transcript table with relative abundance was generated for each sample and compared using principal coordinate analysis of the Bray-Curtis distance. **b** Bray-Curtis distance separating each sample from control (untreated) microbiota was determined and plotted. Data are the means ± S.E.M, with individual data points being represented (*N =* 3). *P < 0.05 compared to the untreated group, determined by a one-way analysis of variance corrected for multiple comparisons with a Bonferroni post-test

That none of the emulsifier tested impacted relative abundance of Enterobacteriaceae, the family that contains numerous pro-inflammatory pathobionts, suggests that the observed increase in the pro-inflammatory potential may preferentially reflect changes in microbiota gene expression rather than species composition (Figure S8). To broadly investigate this possibility, we performed an untargeted microbiota metatranscriptomic analysis in response to our panel of dietary emulsifiers. Total mRNAs were extracted from MBRA samples collected in the middle of the treatment phase (120 h post-inoculation, 48 h after the beginning of emulsifier exposure), rRNA were depleted, and the remaining material was used for library construction and Illumina sequencing. Transcript were assigned to a function and a bacterial origin using the UniRef50 database, and principal coordinate analysis of the Bray-Curtis distance was used to globally assess each compound’s impact on microbiota gene expression. Visualizing the resulting matrices by PCoA analysis revealed clear treatment-based clustering, indicating that most of the 20 food additives tested impact the metatranscriptome. To quantitate the extent of such global changes in gene expression, we calculated Bray-Curtis distances separating emulsifier-treated from untreated microbiota. This approach indicated statistically significant impacts on the transcriptome in response to P80, maltodextrin, propylene glycol alginate, kappa and lambda carrageenans, gum arabic, guar gum, DATEM, and glyceryl stearate (Fig. 7). Assessing alteration in the metatranscriptome based on the number of genes strongly and significantly altered indicated that impacts were most pronounced in response to P80, maltodextrin, iota and kappa carrageenan, guar gum, and locust bean gum (Figure S9). Interestingly, all the emulsifiers used in this study but HPMC had a stronger ability to inhibit, instead of promoting, gene expression (Figure S9). Metatranscriptome analysis by biological processes (Figure S10) and molecular functions (Figure S11) revealed that the significantly altered genes are broad, with every emulsifier used here able to alter the expression of specific pathways, with some alterations being shared between kappa and lambda carrageenans (Figures S10 and S11). Together, these results indicate that some compounds impacted both microbiota composition and gene expression (P80, maltodextrin, propylene glycol alginate, kappa and lambda carrageenan, gum arabic, locust bean gum), while others impacted microbiota gene expression without altering significantly the microbial community structure (DATEM and glyceryl stearate).

**Fig. 8:**
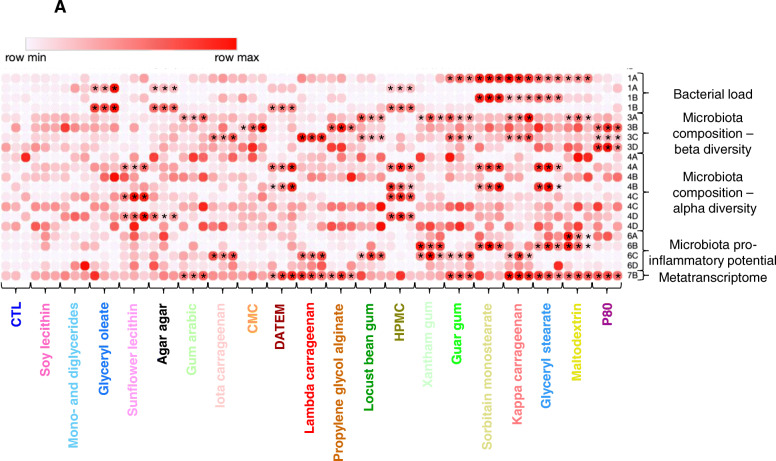
Global compositional and functional effects of dietary emulsifiers on the human microbiota. Heatmap visualization of the impact of dietary emulsifiers on bacterial density (presented in Fig. 1), microbiota composition (beta (presented in Fig. 3) and alpha (presented in Fig. 4) diversity), expression of microbiota-derived pro-inflammatory molecules (presented in Fig. 6), and metatranscriptome (presented in Fig. 7) the human microbiota. *P < 0.05 compared to the untreated group, determined by a one-way analysis of variance corrected for multiple comparisons with a Bonferroni post-test. Emulsifiers are listed from the lowest (left side) to the highest (right side) effect on microbiota composition and function

To conclude, investigation of the effects of 20 commonly used dietary emulsifiers on the intestinal microbiota composition and function reveals that most tested compounds had seemingly negative impacts on microbiota yet some, namely soy lecithin and mono- and diglycerides did not have a discernable impact based on the measurements performed in this study (Table 2 and Fig. 8). Glyceryl oleate and sunflower lecithin only impacted bacterial density and alpha diversity, respectively, and agar agar, gum arabic, and iota carrageenan only impacted one or two parameters measured in a reversible manner (Table 2 and Fig. 8). CMC impacted microbiota composition in a non-reversible manner, while DATEM and lambda carrageenan impacted three parameters in a reversible manner. Propylene glycol alginate impacted metatranscriptome and microbiota composition in a non-reversible manner (Table 2 and Fig. 8). Locust bean gum, HPMC, guar gum, and kappa carrageenan impacted various parameters in a reversible manner, while xantham gum, sorbitan monostearate, glyceryl stearate, maltodextrin, and P80 impacted various microbiota parameters, both compositionally and/or functionally, in a non-reversible manner (Table 2 and Fig. 8). These results suggest that particular caution should be employed for these latter compounds, and suggest priorities for further in vivo testing of these emulsifiers broadly used by the food industry.

**Table 2:**
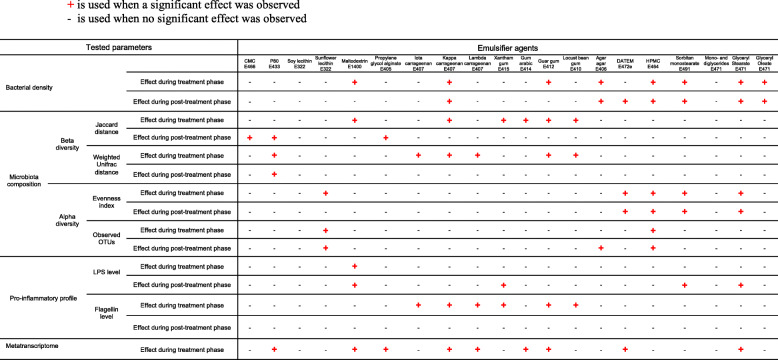
Global compositional and functional effects of dietary emulsifiers on the human microbiota. + correspond to a significant effect

## Discussion

Chronic inflammatory diseases, such as metabolic syndrome and IBD, are associated with disturbance of the composition and the function of the human gut microbiota, with accumulating evidence demonstrating the role played by this community in disease establishment and chronicity. That the prevalence of these disorders has markedly increased amidst relatively constant human genetics indicates a role for non-genetic (i.e., environmental) factors including those that might impact gut microbiota and/or the intestine’s ability to manage this microbial ecosystem. Accordingly, components of modern diets, especially non-absorbed additives that transit the colon and directly interact with the microbiota and the intestinal mucosa, are potential candidates to promote these chronic diseases. Dietary emulsifiers, specifically the synthetic compounds CMC and P80, directly detrimentally impact the intestinal microbiota in ways suggested to promote various inflammatory diseases [5, 11, 14]. Such observations suggest that greater consideration needs to be paid to the potential health hazards of this class of food additive, which might reveal strategies to reformulate some processed food to improve their healthfulness.

Toward the goal of more broadly considering potential impacts of dietary emulsifiers as a class of food additives, we utilized a high-throughput MBRA model of human microbiota in order to screen the impact of 20 commonly used emulsifiers. The use of this model largely recapitulated our results from the more complex but cumbersome Simulated Human Intestinal Microbiota Ecosystem (SHIME) model that showed CMC and P80 directly impacted on microbiota composition and gene expression [11]. Specifically, although neither CMC nor P80 significantly impacted bacterial density, both significantly impacted microbiota composition in a non-reversible manner, as assessed by measurement of the Jaccard distance. P80 also impacted the composition of microbiota as assessed by weighted UniFrac measurement, and also triggered significant metatranscriptome alteration, indicating that P80 had a long-lasting impact on both microbiota composition and gene expression, and further highlighting some differences between the impacts triggered by CMC and P80. In contrast, several of the newly tested compounds, such as sorbitan monostearate and glyceryl stearate, significantly increased microbial density that persisted throughout the post-treatment period studied. The addition of agar agar, DATEM, HPMC, and glyceryl oleate to MBRA microbiotas resulted in a significant non-reversible reduction in bacterial density. This reduction in bacterial density associated with a tendency of these additives to induce a non-reversible decrease in microbiota richness, as observed for DATEM, HPMC, sorbitan monostearate, and glyceryl stearate exposure. In the case of glyceryl stearate, such reduced diversity resulted in a lasting increase in LPS, suggesting that despite lower amounts of total bacteria, the change in composition made the microbiota more able to activate pro-inflammatory signaling. Metatranscriptome analysis did not reveal any gene that could explain the increased levels of bioactive LPS and flagellin observed following treatment with select compounds. This suggests that select emulsifiers may play a role in the release of these pro-inflammatory molecules without impacting the expression of genes involved in their synthesis per se. Thus, future investigations are required to identify microbiota members driving increases in the bioactive level of LPS and flagellin and, moreover, discern underlying mechanisms.

Some of the strongest effects we observed were from maltodextrin, which is not classified as an emulsifier by regulatory agencies but yet have emulsifying properties which impacts food surface characteristics [12]. Maltodextrin impacted several parameters we tested including microbiota density, composition, gene expression, and, perhaps consequently, expression of pro-inflammatory molecules. Such results are consistent with accumulating evidences demonstrating the detrimental impact of maltodextrin on the intestinal environment [9, 10, 30]. However, it should be pointed out that this polysaccharide is thought to be very quickly digested to glucose and absorbed in the small intestine and thus may never have the direct impacts with colon bacteria that we sought to model. Thus, discernment of whether the impacts observed really relate to maltodextrin’s impacts in vivo requires further investigation.

Among the newly tested class of emulsifiers, carrageenan and gum compounds showed notable impacts on both the composition and the function of microbiota, characterized by an elevated expression of pro-inflammatory molecules. Carrageenans (E407) are a group of gel-forming and viscosifying polysaccharides extracted from some species of seaweeds [31]. In this study, we tested three classes of carrageenan, i.e., kappa, iota, and lambda, which chemical structure mainly differs in the number and the position of ester sulfate groups and also in the content of anhydro-galactose [31]. These carrageenans compounds are also characterized by different composition and degree of sulfation at specific locations in the polymer. In food industry, kappa carrageenan is used in order to yield firm gels due to the presence of potassium ions, while iota carrageenan forms soft elastic gels especially in the presence of calcium ions. Unlike kappa and iota, lambda carrageenan is a non-gelling polysaccharide mainly used as a thickener especially due to the presence of three sulfate groups per two galactose molecules. Our results showed that, among these polysaccharides, kappa carrageenan appeared to have the most drastic detrimental impact on the intestinal microbiota, with non-reversible alterations in bacterial density, microbiota composition, and an increased expression of pro-inflammatory molecules. Importantly, with the expression and function of TLR signaling being tightly regulated in the gastro-intestinal tract [32], it remains important to investigate the functional consequences of such increase in pro-inflammatory molecules using in vivo models. Moroever, iota and lambda harbor less effects on the microbiota. Although the detrimental impact of carrageenan compounds on intestinal microbiota has been previously reported in several studies [31, 33–35], kappa carrageenan seems to be especially involved in intestinal inflammation and development of IBD [36, 37], which is in accord with the observation made here on the human microbiota composition and function. That these carrageenan coumpounds are reported to have a detrimental effect on the intestinal epithelium, it remains important to discern between host-effects and microbiota-effects in carrageenan-induced intestinal inflammation [36, 38].

Gum compounds, which are exopolysaccharides, are derived from different sources including microbial origin (xantham gum is derived from the *Xanthonomas campestris* bacterium), or vegetal origin such as gum arabic, guar gum, and locust bean gum which are derived from plants belonging to the plant family *Leguminosae* [39]. These compounds are characterized by different chemical structures and compositions, and their impacts on gut microbiota composition and function are consequently heterogeneous, with guar gum and xantham gum harboring striking detrimental effect, with alterations in bacterial density, composition, as well as an increased expression of pro-inflammatory molecules. Locust bean gum showed important detrimental effects while gum arabic appears to have modest effects on the gut microbiota.

Taxonomic profiling demonstrates profound alterations in microbiota composition at the order and genus levels. Such alterations were characterized by a significant decrease of Lactobacillales members, including *Streptococcus* genus, upon treatment with numerous emulsifiers, including CMC, P80, lecithin compounds, DATEM, and gum and glyceryl compounds. In addition, a remarkable decrease of Clostridiales order, especially *Faecalibacterium* genus, was observed upon treatment with P80, iota carrageenan, HPMC, and mono- and diglycerides. Importantly, previous studies demonstrated a significant reduction of *Faecalibacterium prausnitzii* in IBD patients [29, 40, 41], and this bacterium has been reported to harbor anti-inflammatory properties [42–44]. Moreover, a significant increase in Bacteroidales order was observed upon treatment with kappa carrageenan, lambda carrageenan, DATEM, and glyceryl stearate. In contrast, we observed that 8 of the tested compounds (maltodextrin, gum arabic, guar gum, locust bean gum, DATEM, sorbitan monostearate, glyceryl stearate, and glyceryl oleate) reduced the relative abundance of Verrucomicrobiales, albeit not reaching statistical significance (*p* = 0.06), which was entirely driven by *Akkermansia* genus, known to play a role in numerous inflammatory diseases [45–47] (Figs. 5 and S8). A similar decrease was previously detected in mice upon consumption of guar gum compared to mice fed a fiber-free diet [48], and this result is also in accordance with previous studies demonstrating that a reduced abundance of *Akkermansia* is detected in IBD patients, especially patients with ulcerative colitis, contrary to healthy individuals [49, 50]. Furthermore, a high abundance of *Akkermansia* was shown to be associated with a healthier metabolic status [47], which further suggests a protective role played by this bacterium against chronic inflammatory diseases [45–47], although other studies suggest a more complex and debatable role played by this bacterium [51–53]. It is important to note that our study utilized a single healthy donor, and future studies are warranted to study inter-individual variations in response to emulsifier exposure, as well as to study the impact of these compounds on microbiota from individuals with pre-existing dysbiosis. Moreover, since dietary habits were not investigated on this single healthy donor, the amount of dietary emulsifier consumed prior to feces donation is unknown and could have impacted the in vitro responses observed here, although we presume the 3-day pre-treatment period was sufficient to eliminate any remaining compounds present.

While one might presume that any compound with detergent-like chemical properties, i.e., all emulsifiers, would significantly impact a complex microbial community, in fact, we observed that some of the emulsifiers we tested, namely soy lecithin and mono- and diglycerides, did not drive microbiota dysbiosis in the MBRA model. Yet, detrimental impacts were observed in response to similar compounds. For example, soy lecithin and sunflower lecithin (E322) are derived from lecithin compound which is a mixture of acetone-insoluble phospholipids and other minor substances such as triglycerides and carbohydrates [54]. It has been reported that sunflower lecithin is a non-GMO (non-genetically modified organisms) byproduct and was suggested as an alternative to soybean lecithin [55, 56]. However, we observed here that gut microbiota was more detrimentally impacted by sunflower lecithin, which significantly induced increased levels of FliC during the treatment phase compared with soy lecithin (*p* = 0.0069). This pro-inflammatory effect of sunflower lecithin could be due to its content of omega-6 polyunsaturated fatty acids, previously demonstrated to induce inflammation [57, 58]. Mono- and diglycerides (or glyceryl monostearate), like glyceryl oleate and glyceryl stearate (E471), are derived from monoglyceryl monoesters which are structurally constituted of the esterification products of glycerin and carboxylic acids, mainly including fatty acids [59]. While both mono- and diglycerides and glyceryl oleate did not show significant effects on the human gut microbiota, we surprisingly observed a remarkable detrimental impact of glyceryl stearate on the human microbiota. This suggests that compounds with similar structure and chemical composition can nonetheless have a broadly diverse impact on the gut microbiota composition and function. While these observations beg further study, that some emulsifiers did not drive dysbiosis is nonetheless heartening as it supports the possibility of developing healthier processed foods.

## Conclusion

To conclude, most, but not all, emulsifiers tested here impacted human intestinal microbiota in the MBRA model. Importantly, various food additives are very often used in combination, and it remains important to investigate their effects in combination, especially for compounds with different impacts on the human microbiota. Thus, while future studies are warranted, these data nonetheless importantly suggest that clinical trials are needed so as to reduce the usage of the most detrimental compounds and to favor the use of emulsifying agents with no or low impact on the microbiota [60].

## Supplementary Information

**Additional file 1: Figure S1.** MiniBioReactor Array (MBRA) system and experiment outline. **A**: Overview of the MBRA system installed within an anaerobic chamber. **B**: MBRA system after inoculation and stabilization with human microbiota. **C:** Experimental plan used and schedule of samples collection. **D:** Microbiota stability after MBRA inoculation with human feces. At each time points, the number of unique OTU per 15,000 sequences is represented. Values are mean +/- S.E.M., N = 3. **Figure S2.** Example of the data presentation used in this study. In order to account for the high number of time points analyzed (17) during the three phases of the MBRA experiments (pre-treatment, treatment and post-treatment), data of bacterial density, alpha and beta diversity analysis of microbiota composition and pro-inflammatory potential were processed and presented as exemplified here for the Jaccard measurement of beta diversity of microbiota composition. **A**. Principal coordinate analysis at each individual time points. **B**. Histograms of Jaccard distance separating control samples from every other condition – including control themselves. **C-D**. These various time points were subsequently combined in a XY representation with two normalizations steps : the distance separating control samples from themselves were normalized as 1 (**C**) in order to account for inter-chambers and day-to-day variations, and the distance observed at the 24 h time point were normalize as 1 (**D**) in order to account for pre-treatment inter-chambers variations. **E**. Finally, area under the curve was determined for the treatment phase (72 h > 216 h) and the post-treatment phase (216 h > 274 h) in order to present, for both phases, the global impact of emulsifier exposure on microbiota composition and function. Data are the means +/- S.E.M (*N =* 3). *P < 0.05 compared to untreated group, determined by a one-way analysis of variance corrected for multiple comparisons with a Bonferroni post-test. **Figure S3.** Impact of dietary emulsifiers on bacterial density. Bacterial density was assessed by qPCR during the treatment (96 h, panel **A** and 216 h, panel **B**) and the post-treatment phases (274 h, panel **C**). Data are the means +/- S.E.M, with individual data points being represented (*N =* 3), after normalization of the control group to 1. *P < 0.05 compared to untreated group, determined by a one-way analysis of variance corrected for multiple comparisons with a Bonferroni post-test. **Figure S4.** Impact of dietary emulsifiers on microbiota composition. Microbiota composition was analyzed by 16S rRNA gene sequencing and beta diversity was computed through QIIME2 pipeline using the Jaccard matrix (**A-C**) and the weighted UniFrac distance (**D-F**) during the treatment (96 h, panel **A** and **D** and 216 h, panel **B** and **E**) and the post-treatment phases (274 h, panel **C** and **F**). Data are the means +/- S.E.M, with individual data points being represented (*N =* 3), after normalization of the control group to 1. *P < 0.05 compared to untreated group, determined by a one-way analysis of variance corrected for multiple comparisons with a Bonferroni post-test. **Figure S5.** Impact of dietary emulsifiers on microbiota diversity. Microbiota composition was analyzed by 16S rRNA gene sequencing and alpha diversity was computed through QIIME2 pipeline using the evenness index (**A-C**) and the number of observed OTUs (**D-F**) during the treatment (96 h, panel **A** and **D** and 216 h, panel **B** and **E**) and the post-treatment phases (274 h, panel **C** and **F**). Data are the means +/- S.E.M, with individual data points being represented (*N =* 3), after normalization of the control group to 1. *P < 0.05 compared to untreated group, determined by a one-way analysis of variance corrected for multiple comparisons with a Bonferroni post-test. **Figure S6.** Impact of dietary emulsifiers on the expression of microbiota-derived pro-inflammatory molecules. Microbiota-derived expression of pro-inflammatory molecules was analyzed using HEK cells expressing TLR4 or TLR5 in order to quantify bioactive levels of lipopolysaccharide (LPS) (**A-C**) and flagellin (FliC) (**D-E**), respectively, during the treatment (96 h, panel **A** and **D** and 216 h, panel **B** and **E**) and the post-treatment phases (274 h, panel **C** and **F**). Data are the means +/- S.E.M, with individual data points being represented (*N =* 3), after normalization of the control group to 1. *P < 0.05 compared to untreated group, determined by a one-way analysis of variance corrected for multiple comparisons with a Bonferroni post-test. **Figure S7.** Reproducibility of the MBRA in vitro microbiota system. **A**. Microbiota composition was analyzed by 16S rRNA gene sequencing and taxonomic analysis were computed through QIIME2 pipeline at the order level, for all the time points of the control / untreated chambers from the three independent experiments. **Figure S8.** Impact of commonly-used dietary emulsifiers on microbiota composition at the order level. Microbiota composition was analyzed by 16S rRNA gene sequencing and taxonomic analysis were computed through QIIME2 pipeline at the order level for the 144 h time point. *P < 0.05 compared to untreated group determined by a one-way analysis of variance corrected for multiple comparisons with a Bonferroni post-test. **Figure S9.** Dietary emulsifiers alter microbial gene expression in the MBRA model. Metranscriptome were analyzed by RNA-seq of MBRA suspension collected at the 120 h time point and are represented as volcano plots of emulsifier-treated samples compared to untreated samples. For each identified transcript, the difference in abundance between the two groups is indicated in log2 fold change on x-axis (with positive values corresponding to an increase in emulsifier-treated group compared with untreated group, and negative values corresponding to a decrease in emulsifier-treated group compared with untreated group), and significance between the two groups is indicated by −log10 q value on the y-axis. Green dots correspond to KEGG identifiers with at least a sixteen-fold decreased abundance in emulsifier-treated group compared with untreated group and with a q value < 0.1. The number in green color in each panel represent the number of green dots. Red dots correspond to KEGG identifiers with at least a sixteen-fold increase abundance in emulsifier-treated group compared with untreated group and with a q value < 0.1. The number in red color in each panel represent the number of red dots. **Figure S10.** Impact of dietary emulsifiers on *in vitro* microbiota metatranscriptomes. Total RNAs were extracted from MBRA suspension collected at the 120 h time point and subjected to RNA sequencing. HUMAnN2 program was used to profile for the abundance of microbial pathways and gene families, and a transcript table with relative abundance was generated for each sample and simplified at the biological processes level. The heatmap represent significantly impacted biological processes in emulsifier-treated compared with control, as determined by a two-way analysis of variance corrected for multiple comparisons with a Bonferroni post-test. **Figure S11.** Impact of dietary emulsifiers on *in vitro* microbiota metatranscriptomes. Total RNAs were extracted from MBRA suspension collected at the 120 h time point and subjected to RNA sequencing. HUMAnN2 program was used to profile for the abundance of microbial pathways and gene families, and a transcript table with relative abundance was generated for each sample and simplified at the molecular functions level. The heatmap represent significantly impacted molecular functions in emulsifier-treated compared with control, as determined by a two-way analysis of variance corrected for multiple comparisons with a Bonferroni post-test. *N* = 3.

## Data Availability

Unprocessed sequencing data are deposited in the European Nucleotide Archive under accession number XXXXXX.

## Abbreviations

CMC: Carboxymethylcellulose
DATEM: Diacetyl tartaric acid ester of mono- and diglycerides
HEK cells: Human embryonic kidney cells
HPMC: Hydroxypropyl methyl cellulose
IBD: Inflammatory bowel diseases
MBRA: MiniBioReactor Arrays
P80: Polysorbate 80
