# Supplementary figures and images for: Direct impact of commonly used dietary emulsifiers on human gut microbiota
Source: Microbiome. 2021 Mar 22;9:66. doi: 10.1186/s40168-020-00996-6 (PMC7986288; doi:10.1186/s40168-020-00996-6)

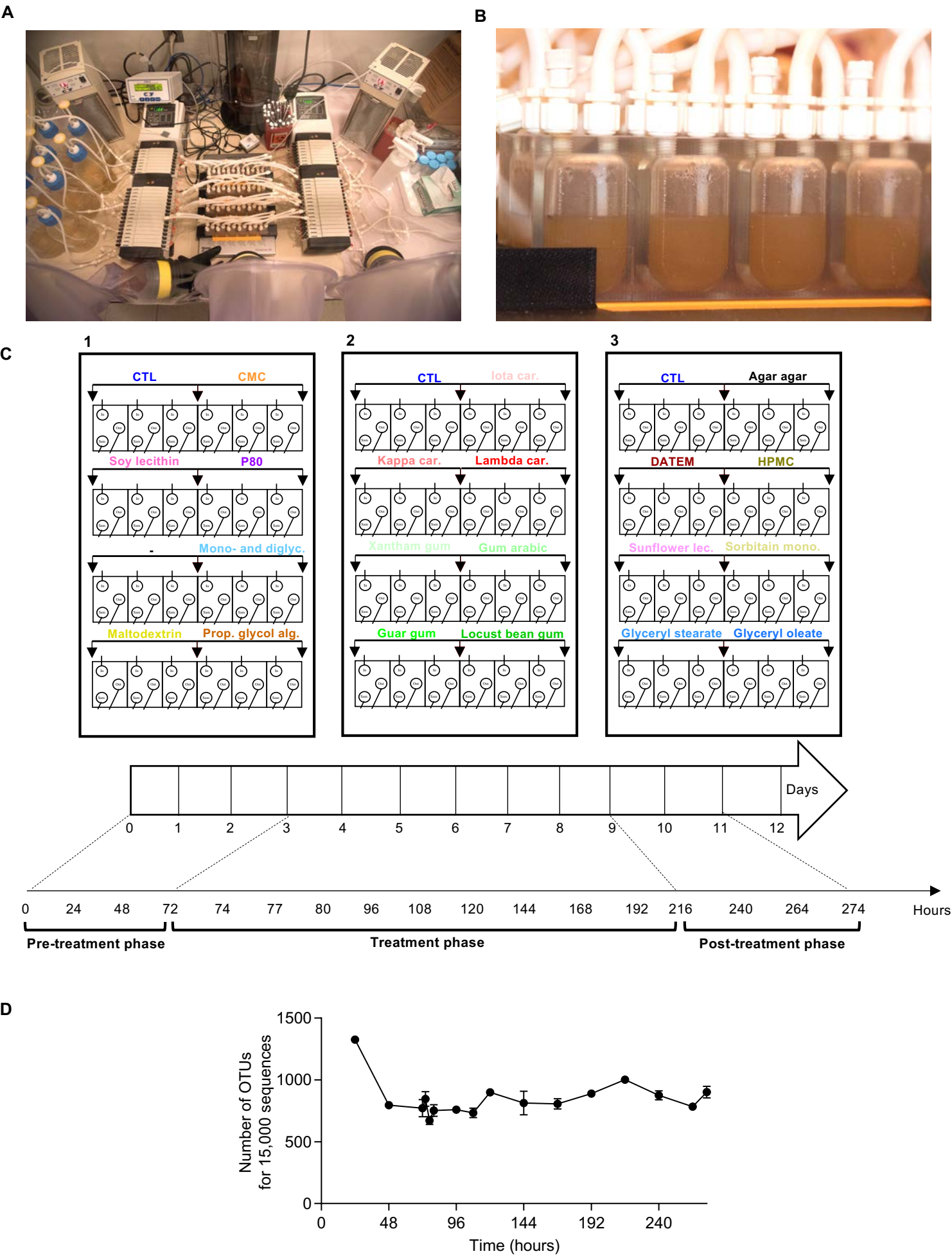

Figure S1.

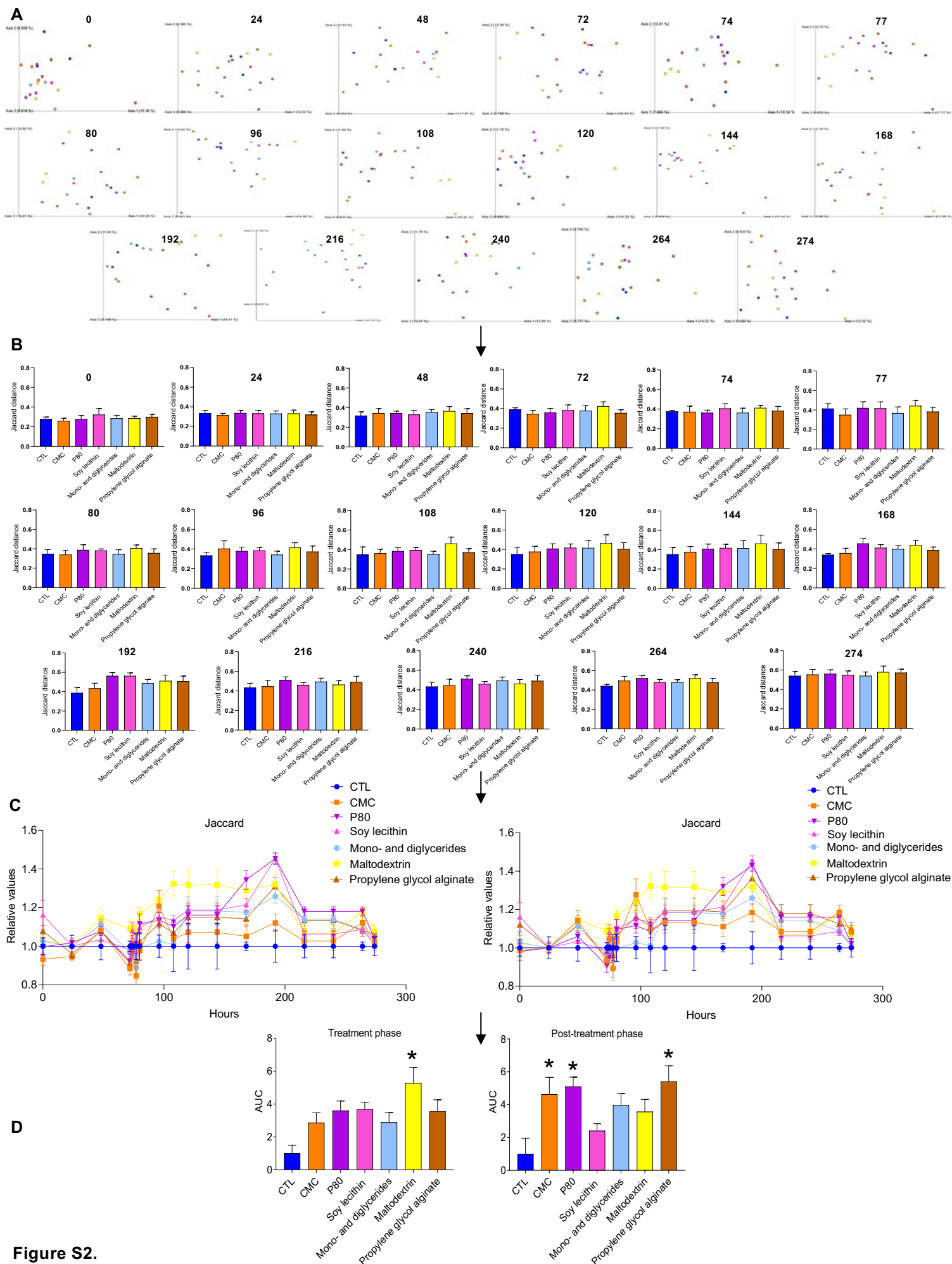

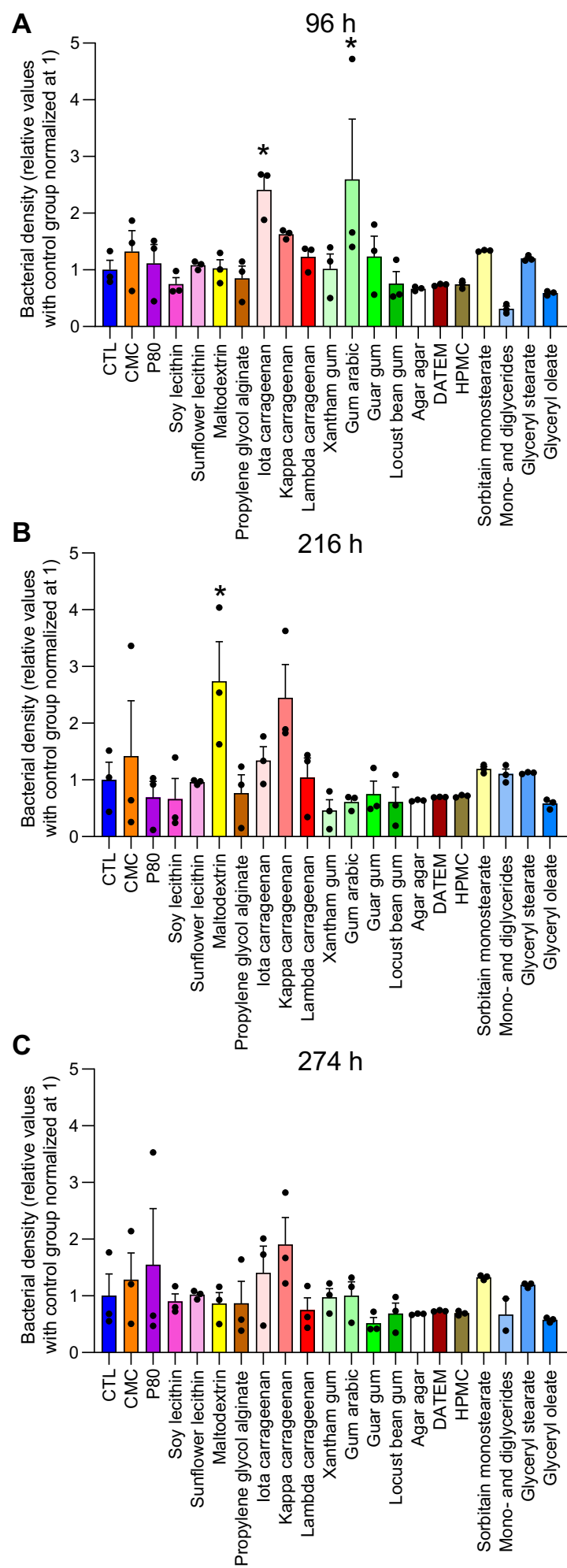

**Figure S3.**

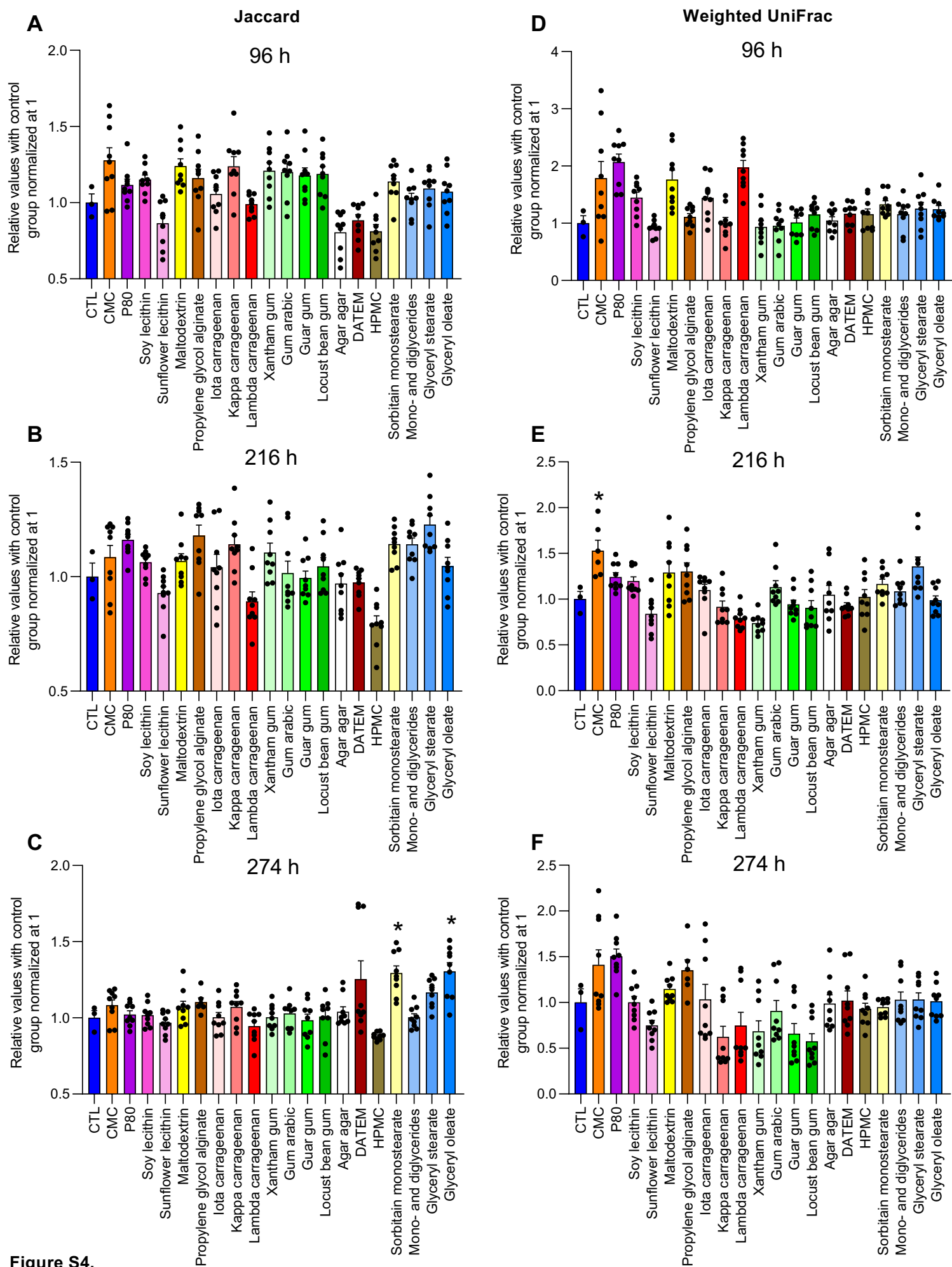

**Figure S4.**

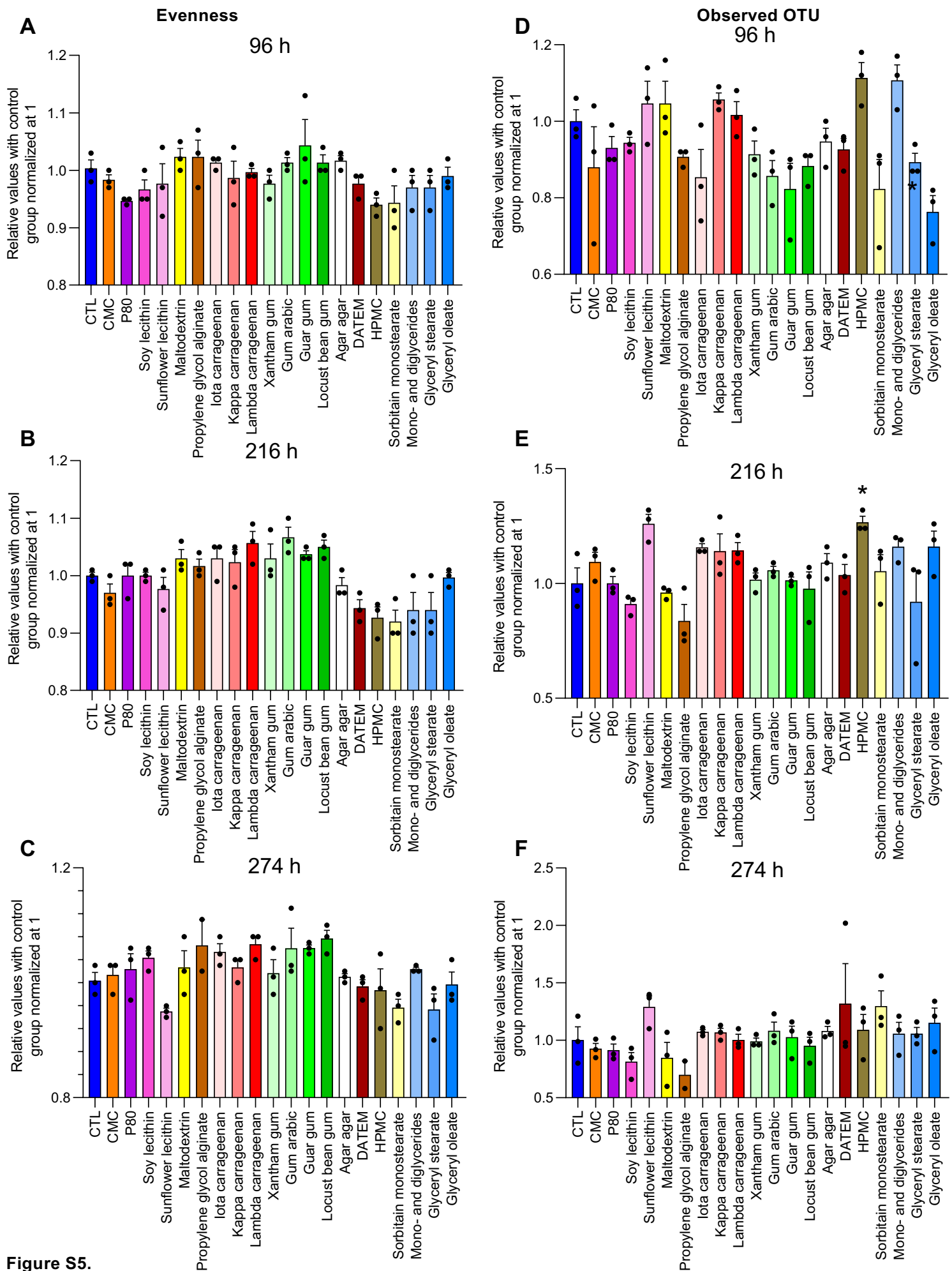

Figure S5.

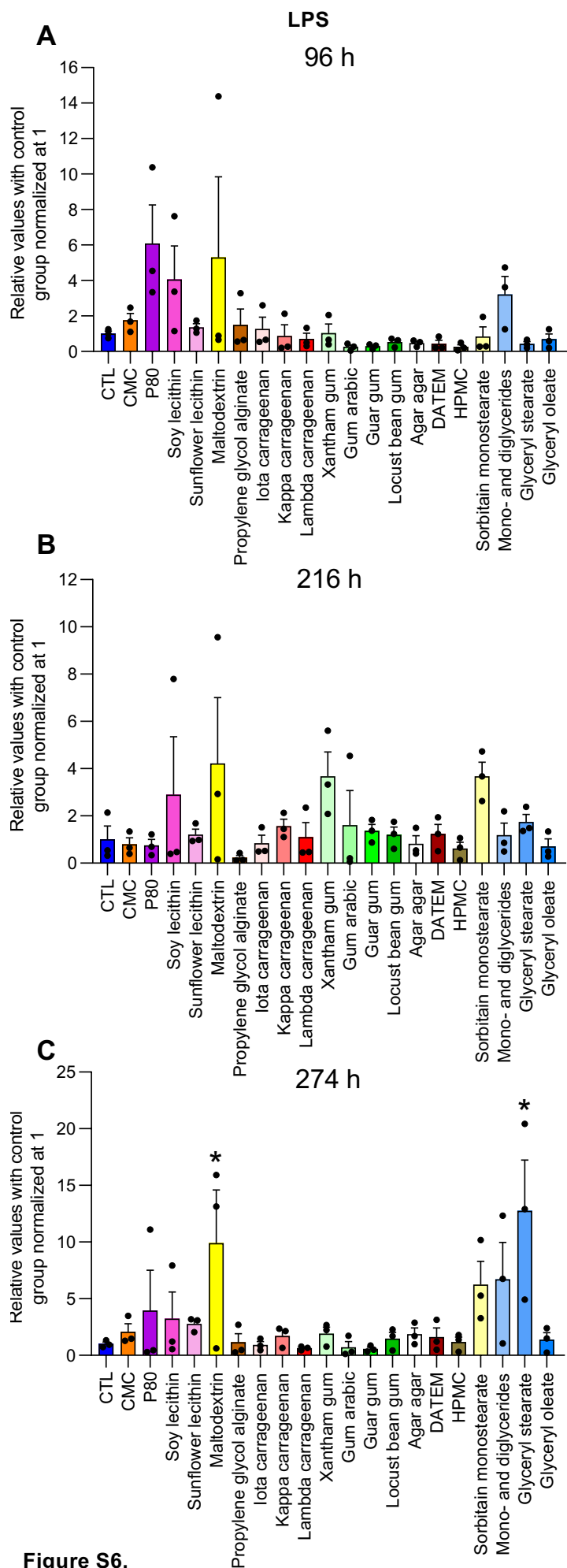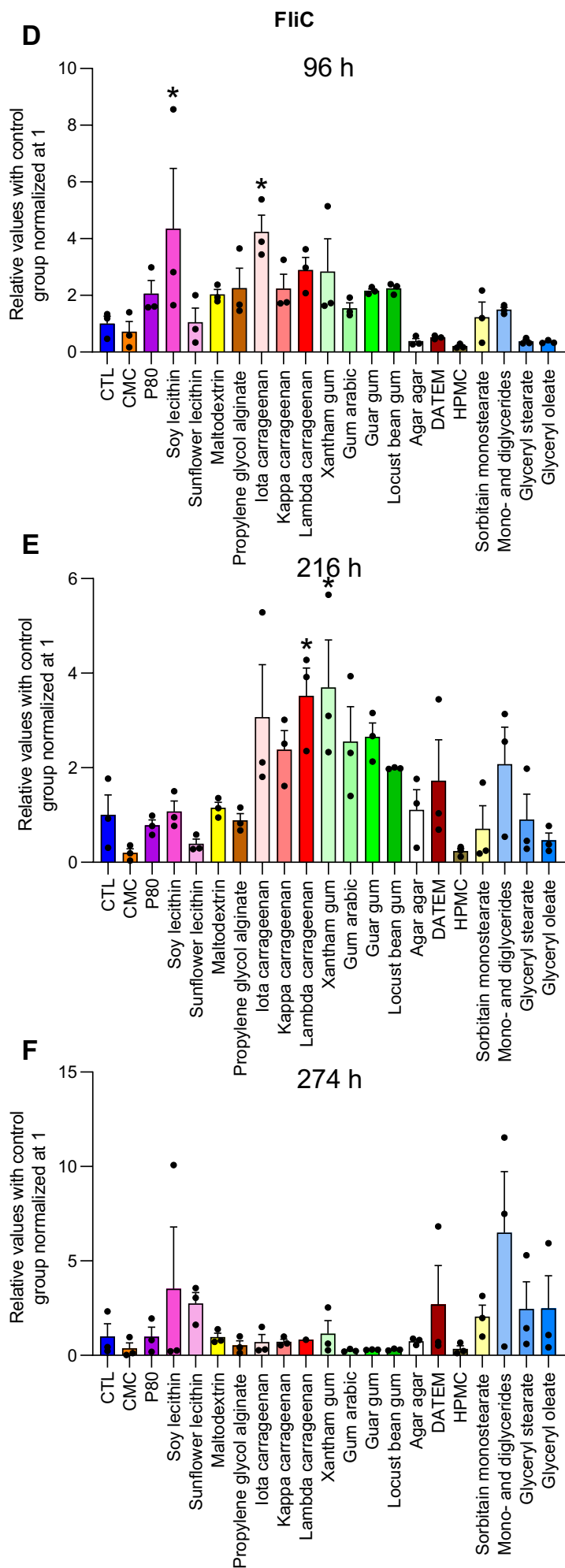

**Figure S6.**

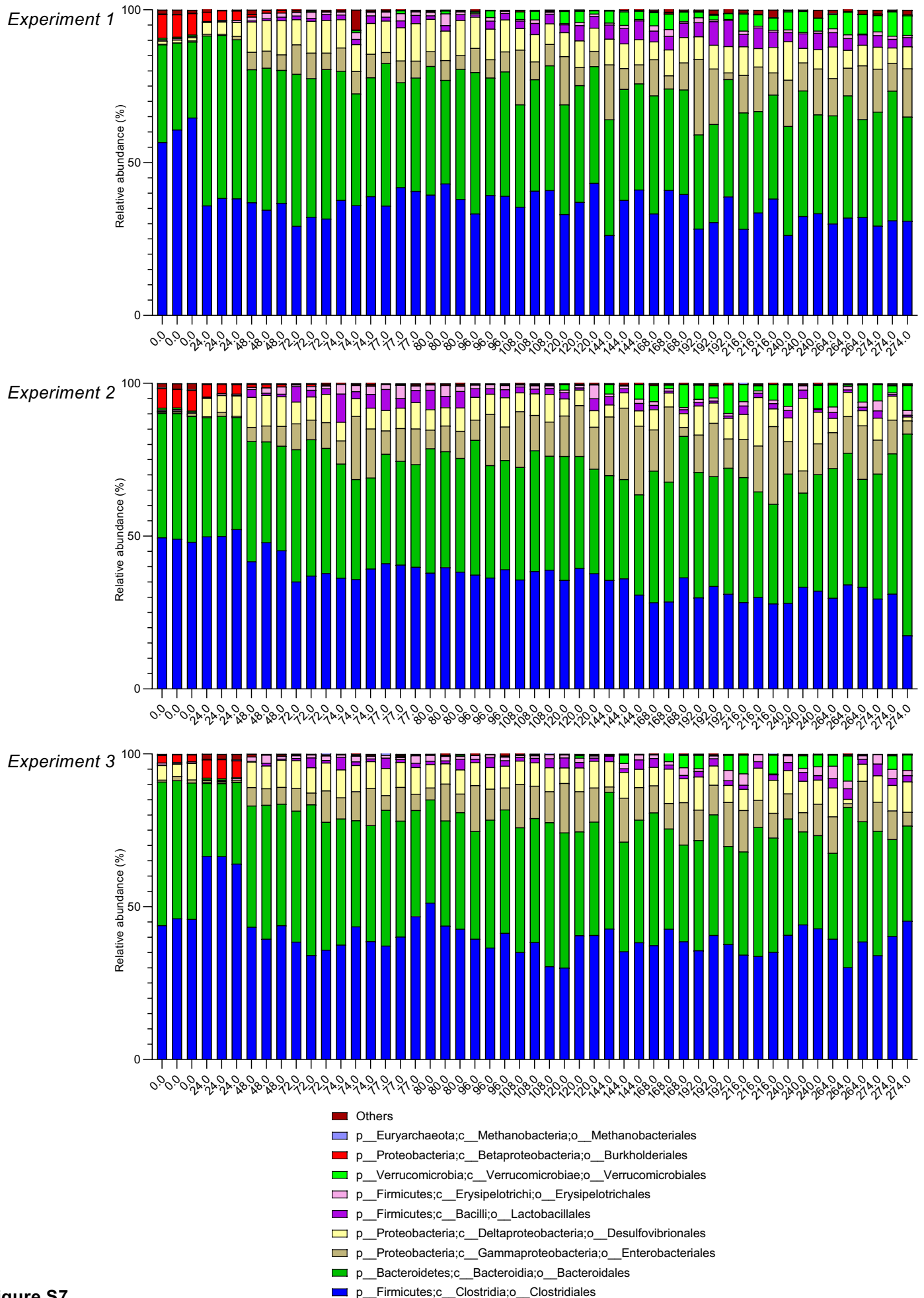

**Figure S7.**

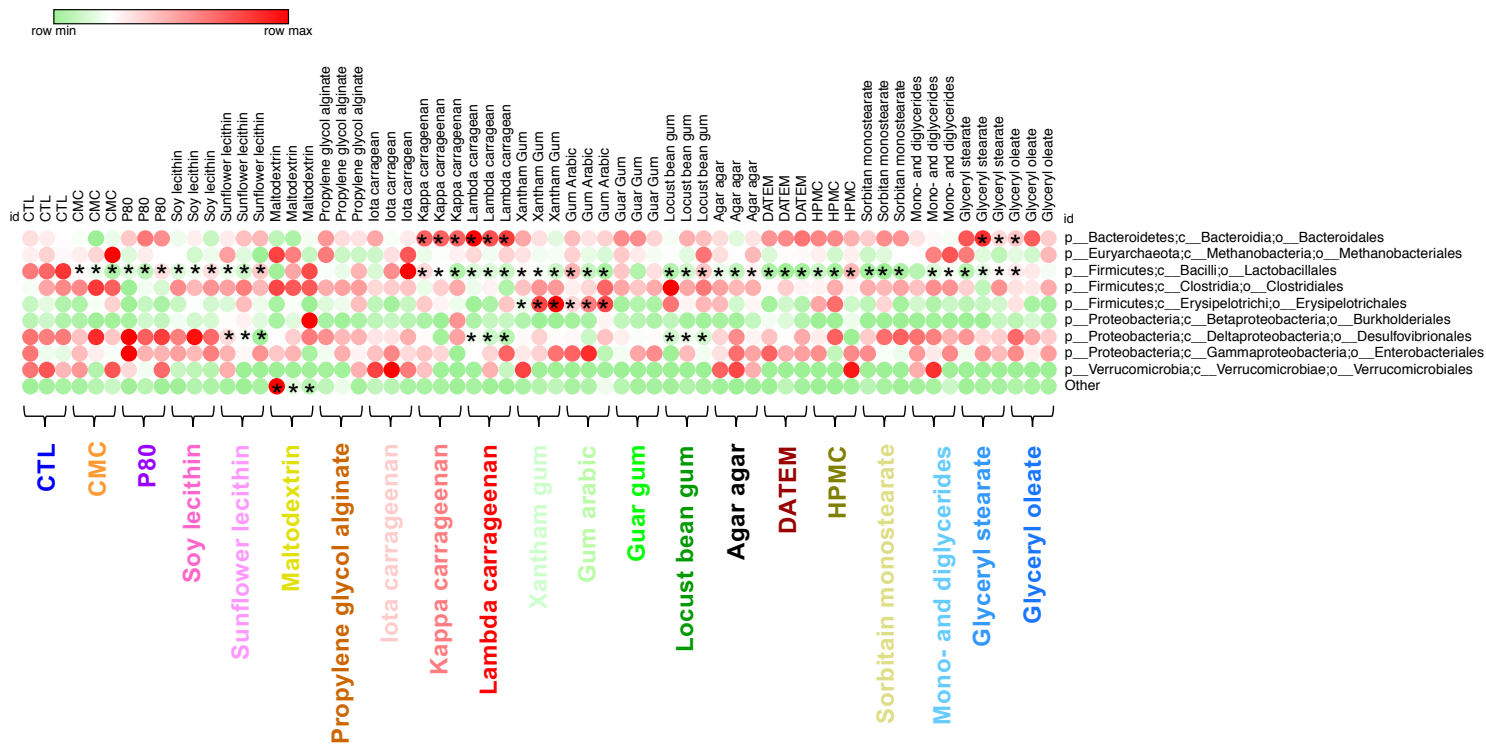

Figure S8.

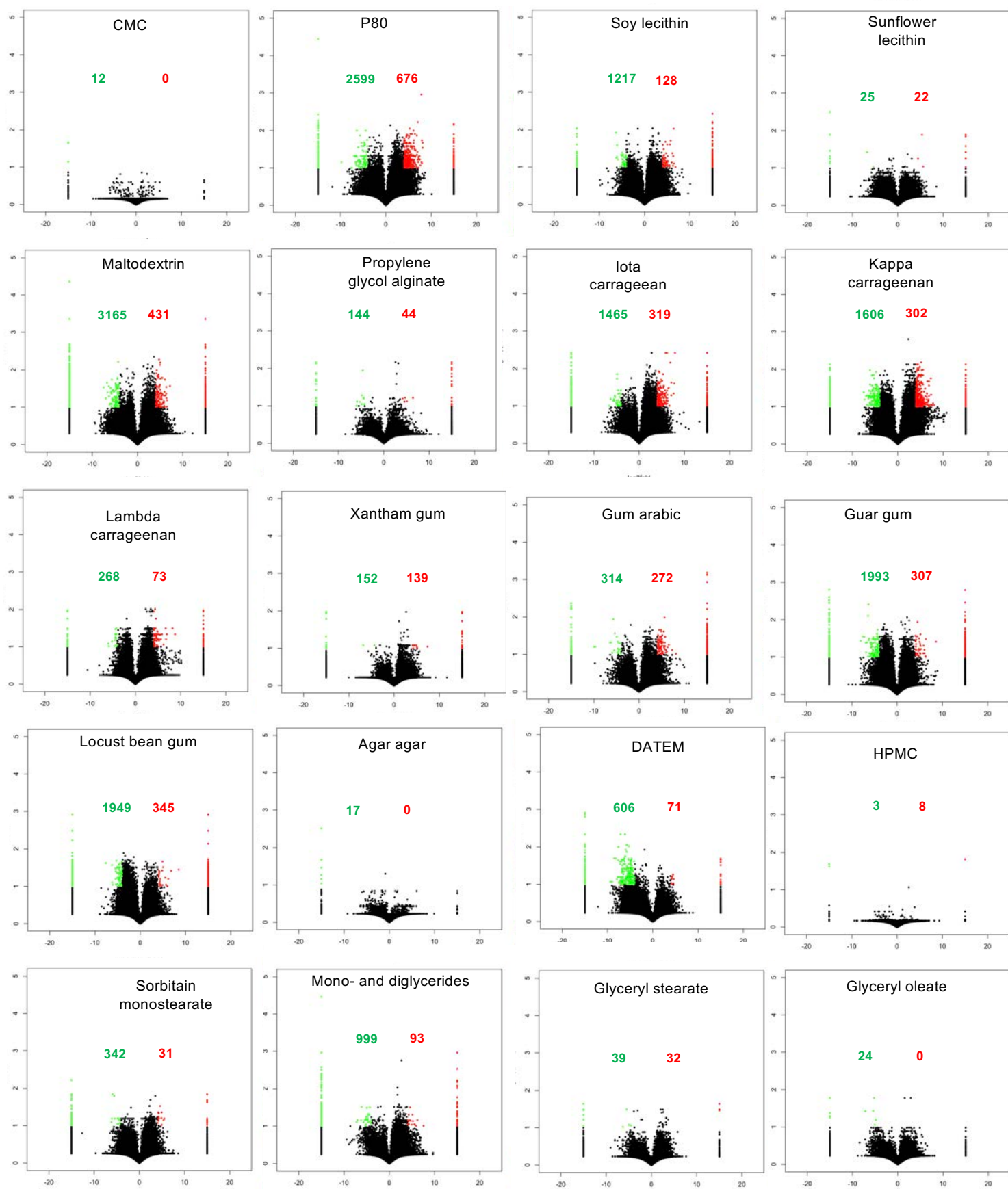

Figure S9.

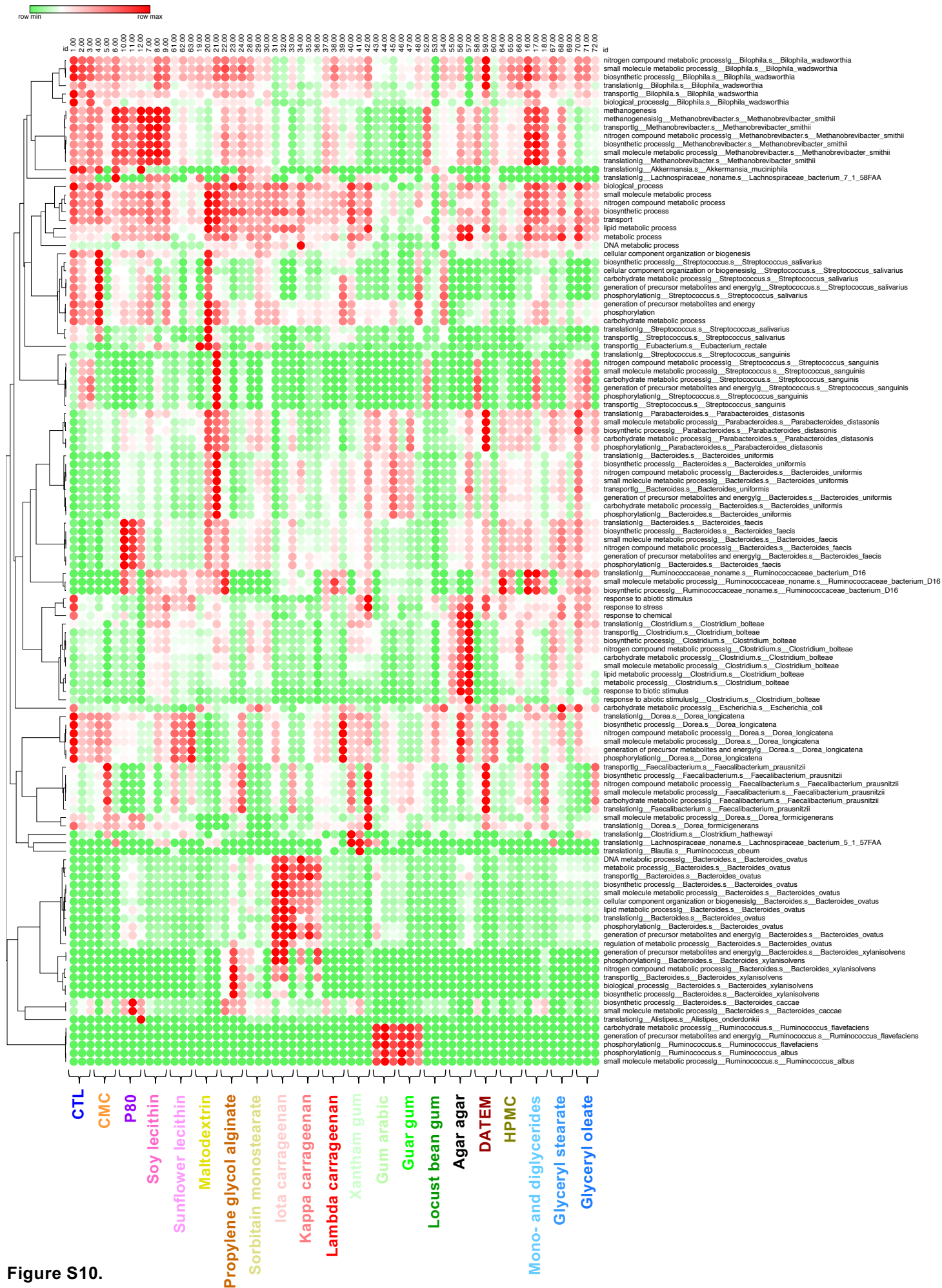

Figure S10.

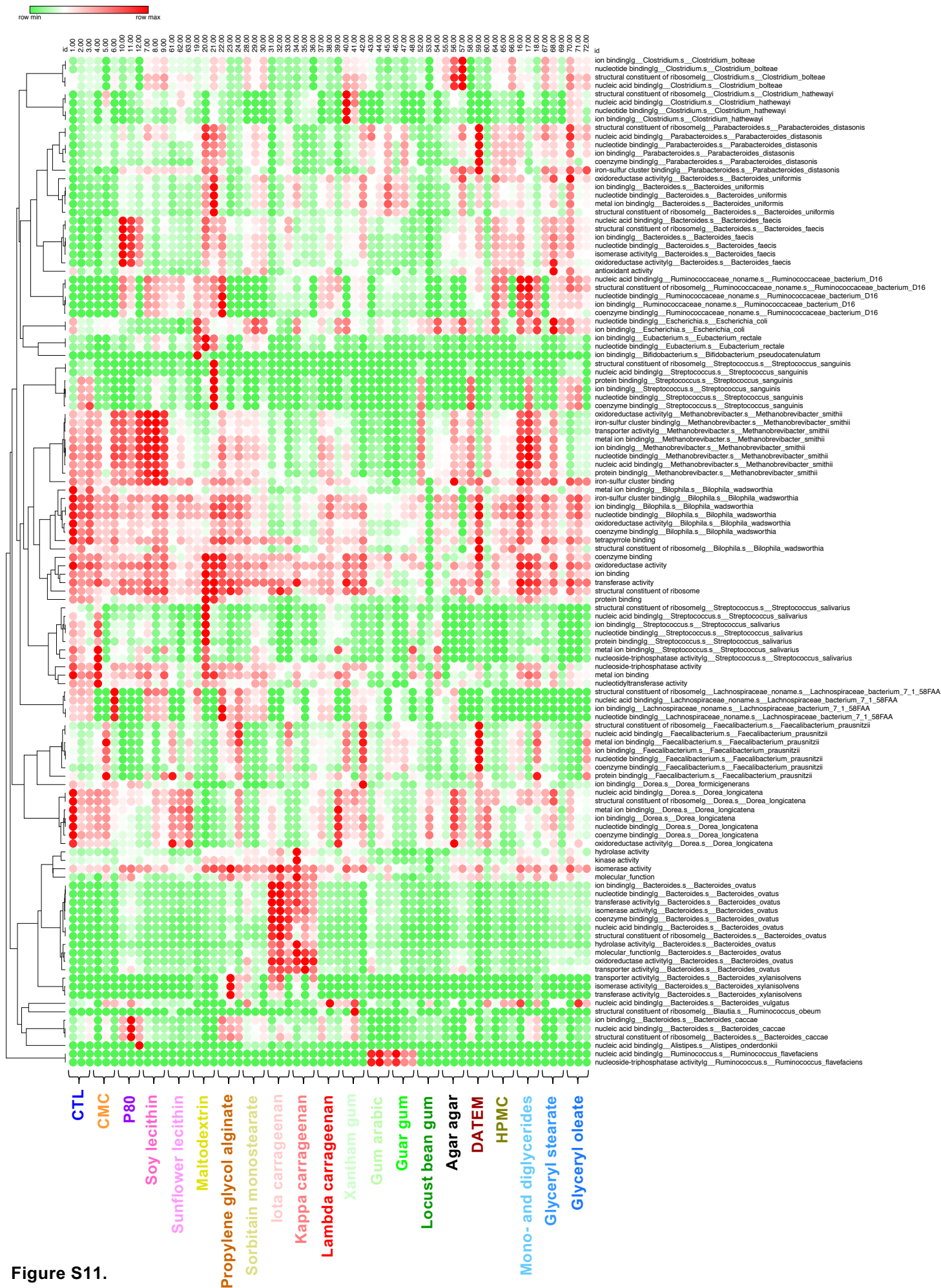

Figure S11.

Supplement: Supplementary file 2 — Additional file 1: Figure S1. MiniBioReactor Array (MBRA) system and experiment outline. A: Overview of the MBRA system installed within an anaerobic chamber. B: MBRA system after inoculation and stabilization with human microbiota. C: Experimental plan used and schedule of samples collection. D: Microbiota stability after MBRA inoculation with human feces. At each time points, the number of unique OTU per 15,000 sequences is represented. Values are mean +/- S.E.M., N = 3. Figure S2. Example of the data presentation used in this study. In order to account for the high number of time points analyzed (17) during the three phases of the MBRA experiments (pre-treatment, treatment and post-treatment), data of bacterial density, alpha and beta diversity analysis of microbiota composition and pro-inflammatory potential were processed and presented as exemplified here for the Jaccard measurement of beta diversity of microbiota composition. A. Principal coordinate analysis at each individual time points. B. Histograms of Jaccard distance separating control samples from every other condition – including control themselves. C-D. These various time points were subsequently combined in a XY representation with two normalizations steps : the distance separating control samples from themselves were normalized as 1 (C) in order to account for inter-chambers and day-to-day variations, and the distance observed at the 24 h time point were normalize as 1 (D) in order to account for pre-treatment inter-chambers variations. E. Finally, area under the curve was determined for the treatment phase (72 h > 216 h) and the post-treatment phase (216 h > 274 h) in order to present, for both phases, the global impact of emulsifier exposure on microbiota composition and function. Data are the means +/- S.E.M (N = 3). *P < 0.05 compared to untreated group, determined by a one-way analysis of variance corrected for multiple comparisons with a Bonferroni post-test. Figure S3. Impact of dietary emuls [file 40168_2020_996_MOESM2_ESM.pdf]
